# Study of PARP inhibitors for breast cancer based on enhanced multiple kernel function SVR with PSO

**DOI:** 10.3389/fphar.2024.1257253

**Published:** 2024-02-02

**Authors:** Haohan Xue, Ruixuan Zhang, Xudong Yan, Ruihan Wang, Peijian Zhang

**Affiliations:** University, College of Computer Science and Technology, Qingdao, China

**Keywords:** breast cancer, PARP inhibitor, quantitative structure-activity relationship (QSAR), support vector regression (SVR), multiple kernel function, particle swarm optimization (PSO)

## Abstract

PARP1 is one of six enzymes required for the highly error-prone DNA repair pathway microhomology-mediated end joining (MMEJ) and needs to be inhibited when over-expressed. In order to study the PARP1 inhibitory effect of fused tetracyclic or pentacyclic dihydrodiazepinoindolone derivatives (FTPDDs) by quantitative structure-activity relationship technique, six models were established by four kinds of methods, heuristic method, gene expression programming, random forester, and support vector regression with single, double, and triple kernel function respectively. The single, double, and triple kernel functions were RBF kernel function, the integration of RBF and polynomial kernel functions, and the integration of RBF, polynomial, and linear kernel functions respectively. The problem of multi-parameter optimization introduced in the support vector regression model was solved by the particle swarm optimization algorithm. Among the models, the model established by support vector regression with triple kernel function, in which the optimal *R* ^2^ and RMSE of training set and test set were 0.9353, 0.9348 and 0.0157, 0.0288, and R^2^_cv_ of training set and test set were 0.9090 and 0.8971, shows the strongest prediction ability and robustness. The method of support vector regression with triple kernel function is a great promotion in the field of quantitative structure-activity relationship, which will contribute a lot to designing and screening new drug molecules. The information contained in the model can provide important factors that guide drug design. Based on these factors, six new FTPDDs have been designed. Using molecular docking experiments to determine the properties of new derivatives, the new drug was ultimately successfully designed.

## 1 Introduction

Breast cancer is a common malignant disease in breast tissue. In 2020, more than 2.26 million people were diagnosed with the disease and about 685 thousand people were killed by it (Sung et al., 2021). Because of the high incidence rate and seriousness of breast cancer, developing new drugs with good therapeutic effects has become a hot research spot. The size of the breast cancer drug development market has totaled 20.2 billion dollars in 2019 and is forecast to grow to 47.7 billion in 2029 (Wilcock and Webster, 2021), which makes breast cancer drug development become one of the biggest disease research fields.

Although new breast cancer drugs have been developed continuously, the efficiency of most drugs has been limited due to side effects and the lack of specificity. Since Bryant et al. proposed the concept of synthetic lethality effect in 2005, the anti-tumor effect of Poly (ADP-ribose) polymerase (PARP) inhibitors has been gradually revealed.

The PARP family, known as diphtheria-toxin-like ADP-ribosyltransferases (ARTDs), is a family of 17 enzymes that share a common catalytic ADP-ribosyl transferase (ART) motif (Zong et al., 2022). PARP can be activated by recognizing DNA fragments of structural damage and then perform base excision repair (Zong et al., 2022). It plays an important role in DNA single-strand break (SSB) repair, programmed cell death regulation, and DNA stability maintenance (Ohmoto and Yachida, 2017). Among the 17 kinds of PARP, PARP1 and PARP2 are the two most important subtypes of the PARP family (Huang and Yin, 2021). And at present, PARP inhibitors mainly inhibit these two subtypes.

PARP1 is one of six enzymes required for the highly error-prone DNA repair pathway microhomology-mediated end joining (MMEJ) (Sharma et al., 2015). When PARP1 is upregulated, MMEJ is increased, causing genome instability (Muvarak et al., 2015). Ordinarily, deficient expression of a DNA repair enzyme results in increased un-repaired DNA damage which leads to mutations and cancer through replication errors. However, the accuracy of MMEJ repair mediated by PARP1 is significantly low. Therefore, it seems that cancer is more likely to occur due to over-expression rather than under-expression (Sharma et al., 2015).

In breast cancer cells, the breast cancer susceptibility gene BRCA1 and BRCA2 genes are significant tumor suppressors for DNA double-strand breaks (DSBs) by homologous recombination (HR) and their mutation easily causes genetic instability and leads to the emergence of tumor cells (Luo et al., 2021).

PARP inhibitors can selectively kill tumor cells with HR function defects caused by RBCA1 and BRCA2 genes mutation by inhibiting PARP activity and leading to DNA repair failure, but have no effect on normal cells, which is synergistic lethal effect (Lord and Ashworth, 2017). PARP inhibitors show good curative effects in breast cancer therapies, which makes the research of PARP inhibitors become a significant field in breast cancer drug research.

In the experiments shown in literature (Wang H. et al., 2020), fused tetracyclic or pentacyclic dihydrodiazepinoindolone derivatives (FTPDDs) were studied as PARP inhibitors, and Pamiparib was found with great effects in inhibiting the activity of PARP1. Therefore, some FTPDDs that have a similar structure to Pamiparib should be deeply studied to find PARP1 inhibitors with better therapeutic effects and fewer side effects. The inhibitory activity on PARP can be evaluated by PARP IC_50_. Therefore, obtaining PARP1 IC_50_ values of FTPDDs is of great importance for screening out PARP1 inhibitors with high biological activity and low toxicity, which will contribute to screening out FTPDDs with good therapeutic effects subsequently. Since the traditional methods of measuring IC_50_ consume a lot of manpower and material resources, quantitative structure-activity relationship (QSAR) technique is used to predict the IC_50_ values of compounds quickly and accurately.

QSAR is a method that uses mathematical models to describe the relationship between molecular structure and certain biological activity (An et al., 2006; Chen and Si, 2021). The basic assumption of QSAR is that the molecular structure of the compound decides its physical, chemical, and biological properties which subsequently decide its biological activity (Roy and Das, 2014). And the biological and chemical properties of compounds are portrayed by molecular descriptors in QSAR model (Jin et al., 2022; LI et al., 2023). Therefore, QSAR can be used to predict the biological activity of new compounds by mathematical models based on precisely selected molecular descriptors (Vilar et al., 2008; Si et al., 2022). QSAR shows a strong ability in new drug research, which optimizes pharmacodynamic characteristics and reduces expensive experiments in the meanwhile. Therefore, QSAR can be used to predict the IC_50_ of PARP1 inhibitors to screen out new potential drug molecules quickly and accurately.

In this study, six QSAR models based on three kinds of methods, heuristic method (HM), gene expression programming (GEP), random forest and support vector regression (SVR) with single, double, and triple kernel function, were established and compared after calculating molecular descriptors to predict the PARP1 IC_50_ of FTPDDs. The single, double, and triple kernel functions were RBF kernel function, the integration of RBF and polynomial kernel functions, and the integration of RBF, polynomial, and linear kernel functions respectively. Among the results of the models, the predictions of the model constructed by SVR with triple kernel function were consistent with the measured values best. The method of SVR with triple kernel function is a big breakthrough and will contribute a lot to designing and screening new drug molecules. Activity prediction and molecular docking experiments were applied to newly designed multiple compounds. The experimental results indicated that the newly designed compounds showed better performance.

## 2 Computational details and theories

### 2.1 Data set

The data set of 57 FTPDDs was collected from literature (Wang H. et al., 2020) and listed in Tables 1–4 according to the main structure of compounds. It included two parts, the structures of 57 FTPDDs and their PARP1 IC_50_ values which were measured by the same experimental method under the same experimental conditions. Using simple random sampling, the original data is randomly divided into training set and test set according to the ratio of 4:1. The data set was randomly separated into a training set of 45 compounds and a test set of 12 compounds. The training set was used to establish the models and the test set was used to evaluate the prediction ability of the constructed models. For multi model regression evaluation, small datasets are susceptible to noise and randomness, and each model may only have a small number of samples for training, resulting in unstable model results. In addition, overfitting is easy to occur if each model has more degrees of freedom to adjust. Therefore, cross validation is a good method to ensure the stability of machine learning models.

**TABLE 1:** Measured and predicted lg (IC_50_) of FTPDDs 1–16. *The compounds of the test set.

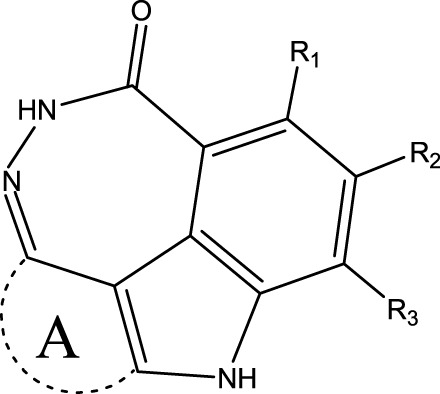											
Compound	R1	R2	R3	A ring	Measured lg (IC_50_)	Predicted lg (IC_50_)					
						HM	GEP	RF	SVR with single kernel function	SVR with double kernel function	SVR with triple kernel function
1*	H	H	H	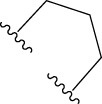	1.0086	1.0480	1.4606	1.0899	1.2200	1.0728	1.2200
2	H	F	H	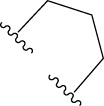	0.7782	1.0871	0.9972	0.8096	0.9442	0.9517	0.9373
3	H	H	F	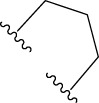	1.3802	1.2053	1.0629	1.0685	1.3561	1.3675	1.3563
4	H	F	H	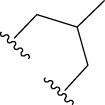	0.6902	0.7277	0.6955	0.6945	0.6659	0.6774	0.6659
5*	H	H	H	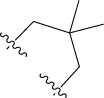	0.7993	0.7922	0.9886	0.8630	0.8140	0.7881	0.8140
6	H	F	H	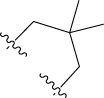	0.7160	0.9350	0.8954	0.6831	0.8124	0.8201	0.7962
7	F	H	H	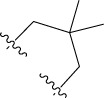	0.9590	1.0478	1.0962	0.9185	0.9346	0.9459	0.9349
8	H	F	H	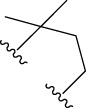	1.4594	1.1364	1.0550	0.9721	1.0666	1.0749	1.0782
9*	H	F	H	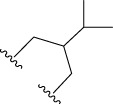	0.5185	0.7771	0.7436	0.6377	0.6878	0.7119	0.6878
10	H	F	H	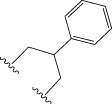	0.7634	0.6953	1.1288	0.8384	0.7878	0.7760	0.7875
11	H	F	H	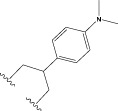	1.4082	1.1383	1.0468	0.9584	1.1978	1.2552	1.2064
12*	H	H	H	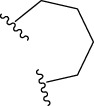	0.8261	0.8217	1.0543	0.8586	0.9331	0.9033	0.9331
13*	H	F	H	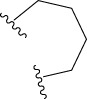	0.8129	0.6934	0.7544	0.8265	0.6387	0.6627	0.6387
14	H	F	H	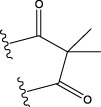	1.1173	1.0121	0.9203	1.0431	1.1414	1.1301	1.0937
15	H	F	H	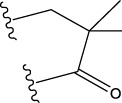	1.0569	1.1801	1.1249	0.9004	1.0812	1.0693	1.0805
16	H	F	H	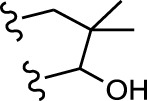	1.0253	0.7540	0.8502	1.0659	1.0016	1.0128	1.0013

**TABLE 2:** Measured and predicted lg (IC_50_) of FTPDDs 17–34. *The compounds of the test set.

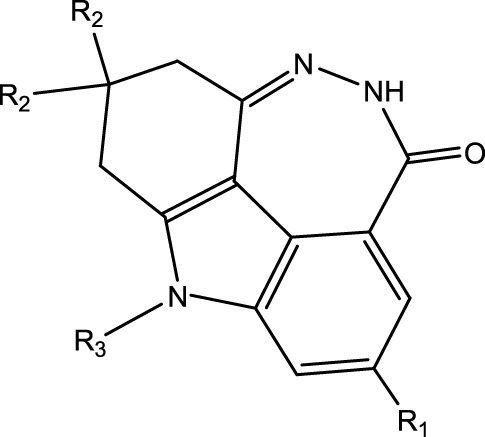										
Compound	R1	R2	R3	Measured lg (IC_50_)	Predicted lg (IC_50_)					
					HM	GEP	RF	SVR with single kernel function	SVR with double kernel function	SVR with triple kernel function
17	H	H	CH_3_	1.1987	1.0851	0.9446	1.2289	1.2230	1.2039	1.2223
18	H	CH3	CH_3_	1.1818	0.9383	0.8455	0.9208	1.1011	1.1694	1.1581
19	H	H	CH_2_CH_2_N(CH_3_)_2_	1.1761	1.1981	1.2457	0.9703	1.2482	1.2250	1.2478
20	H	H	CH_2_CH_2_N(CH_2_CH_3_)_2_	1.1931	0.7624	0.7404	1.0806	0.7965	0.8780	0.8462
21*	H	H	CH_2_CH_2_NBn_2_	2.9031	2.7748	2.2265	1.8054	2.3226	2.5306	2.3226
22	H	H	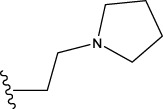	1.0212	0.9255	0.7426	0.9875	0.8878	1.0086	0.9971
23	H	H	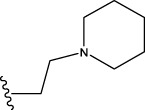	1.0128	1.0061	1.1865	1.0120	0.9883	1.0005	0.9891
24	H	H	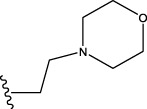	2.5441	2.3479	2.4532	2.4998	2.5196	2.3828	2.4649
25	H	CH3	CH_2_CH_2_N(CH_3_)_2_	0.7404	0.8768	0.8938	0.8139	0.8862	0.9145	0.8930
26	F	CH3	CH_2_CH_3_N(CH_3_)_2_	0.8129	1.0090	0.7815	0.7898	0.8372	0.8260	0.8372
27	H	CH3	CH_2_CH_2_N(CH_2_CH_3_)_2_	0.5185	0.6547	0.8305	0.8301	0.7285	0.6997	0.6425
28	H	CH3	CH_2_CH_2_NBn_2_	2.7076	2.8163	2.6808	2.3985	2.6835	2.6951	2.6842
29	H	CH3	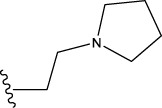	0.8195	0.9021	0.8349	0.8195	0.7954	0.8086	0.7961
30	H	CH_3_	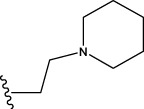	0.4624	0.7450	0.8832	0.7651	0.6989	0.7135	0.6598
31	H	CH_3_	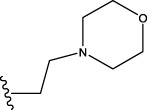	2.2788	2.0614	2.1938	2.2788	2.2725	2.2663	2.2547
32	H	CH_3_	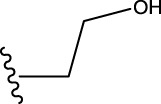	1.3617	0.9713	1.0690	1.2968	1.3373	1.3487	1.3377
33	H	CH_3_	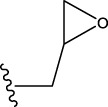	1.4314	1.8920	1.0923	1.4314	1.5439	1.4441	1.4552
34*	H	CH_3_	CH_2_CH_2_ NHCH_3_	0.8451	1.0300	0.9006	0.8653	0.9870	1.0752	0.9870

**TABLE 3:** Measured and predicted lg (IC_50_) of FTPDDs 35–54. *The compounds of the test set.

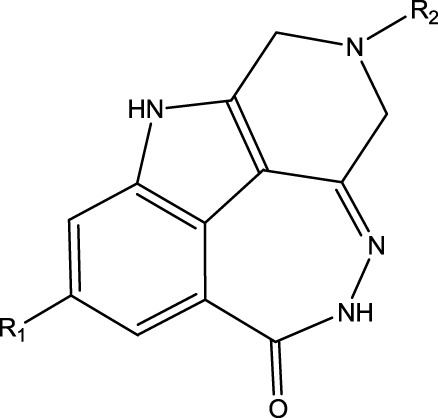									
Compound	R1	R2	Measured lg (IC_50_)	Predicted lg (IC_50_)					
				HM	GEP	RF	SVR with single kernel function	SVR with double kernel function	SVR with triple kernel function
35	H	H	1.1959	1.0914	1.1661	1.0889	1.1718	1.1834	1.1725
36	F	H	0.9294	0.7868	0.7288	1.0242	0.8037	0.9158	0.9056
37	F	CH_3_	0.7559	1.0302	0.8291	0.6576	0.7136	0.7432	0.7318
38	F	CH_2_CH_3_	0.6628	0.7414	0.7206	0.6732	0.6867	0.7006	0.6943
39	F	CH_2_CH_2_CH_3_	0.6532	0.9517	0.8863	0.6829	0.7932	0.7153	0.6835
40*	H	CH(CH_3_)_2_	0.8513	0.9240	0.8963	0.8273	0.7244	0.7829	0.7244
41	F	CH(CH_3_)_2_	0.7076	1.0675	0.8806	0.7076	0.8558	0.8060	0.7999
42*	F	CH_2_ CH_2_CH_2_CH_3_	0.5798	0.7356	0.8124	0.6091	0.7469	0.7113	0.7469
43	H	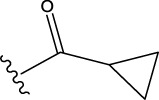	0.5185	0.7679	0.7753	0.5479	0.5685	0.5860	0.5474
44	F	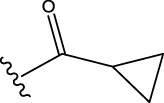	0.3617	0.7052	0.6998	0.3878	0.6200	0.5915	0.5470
45*	F	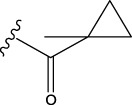	0.9912	0.8559	0.9505	0.6812	0.9069	0.8375	0.9069
46	F	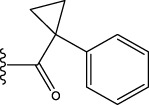	1.4487	1.4786	1.5203	1.3075	1.4730	1.4614	1.4723
47	F	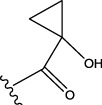	0.6721	1.1065	0.9807	0.6588	0.6962	0.8079	0.6962
48	F	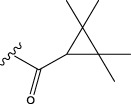	1.2529	0.9978	1.0747	1.0754	1.2285	1.2399	1.2286
49	F	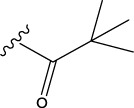	0.8633	0.6836	0.7265	0.8156	0.8391	0.8508	0.8392
50	F	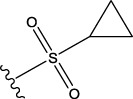	0.9031	0.6838	0.7577	0.7876	0.9277	0.9159	0.9267
51*	F	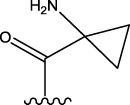	0.9956	0.5959	0.7650	0.9328	0.9403	0.9290	0.9403
52	H	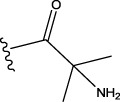	0.8573	0.7521	0.7321	0.8573	0.8336	0.8448	0.8334
53	F	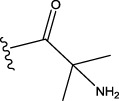	1.0792	0.7365	0.7139	0.8423	1.0121	0.9618	0.9450
54*	F	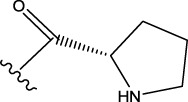	0.8195	1.0923	0.8548	0.5479	1.0716	1.0080	1.0716

**TABLE 4:** Measured and predicted lg (IC_50_) of FTPDDs 55–57. *The compounds of the test set.

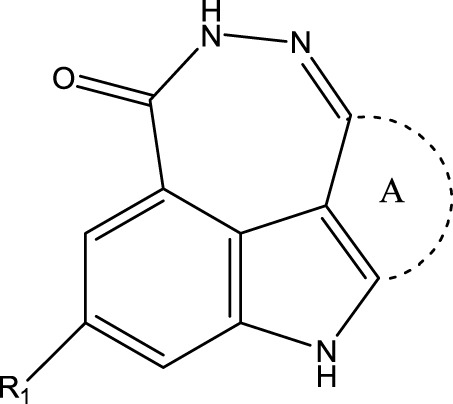									
Compound	R1	A ring	Measured lg (IC_50_)	Predicted lg (IC_50_)					
				HM	GEP	RF	SVR with single kernel function	SVR with double kernel function	SVR with triple kernel function
55	H	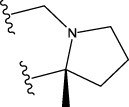	0.9138	0.9582	1.0044	0.9069	0.8895	0.9262	0.9379
56	F	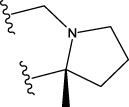	0.7076	0.9856	0.8237	0.7244	0.7324	0.7202	0.7316
57	F	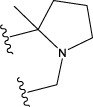	1.8633	1.3363	1.1642	0.8030	1.3451	1.3750	1.4129

In order to test the robustness of the models, K-fold cross-validation was adopted. The procedure involves splitting the original data evenly into K parts, and sequentially using one of these parts as the validation set while using the remaining K-1 parts as training sets. This process is repeated K times, and the average of the K validation results is used to evaluate the model’s performance.

### 2.2 Descriptor calculation

The steps of calculating molecular descriptors of compounds are as follow.

The first three steps include drawing molecular structures of all compounds in ChemDraw software and performing preliminary optimization of the structures of the compounds by MM + molecular mechanics force field and more precise optimization by semi-empirical PM3 (Klein et al., 2006) method successively in HyperChem software (HyperChem, 1994). After the three steps, the lowest energy structures are obtained.

The remaining two steps consist of putting files obtained from HyperChem into MOPAC software (Stewart, 1989) to generate MNO files and then using MNO files as the input of the CODESSA software to calculate five classes of molecular descriptors: constitutional, topological, geometrical, electrostatic, quantumchemical (Wright et al., 1997).

### 2.3 Statistical parameters

The coefficient of determination (denoted *R* ^2^ or *r* ^2^) and root mean square error (RMSE) were used to evaluate the models. *R* ^2^ is a measure of the goodness of fit of a model. In regression, the *R* ^2^ is a statistical measure of how well the regression predictions approximate the real data points. The closer *R* ^2^ is to 1, the more it indicates that the regression prediction fits the data (Glantz and Slinker, 1990). Furthermore, to verify the robustness of the models, the coefficient of determination of K-fold cross-validation (R^2^_cv_) is used in the evaluation of the results, which is the average of all *R* ^2^ values in K cross validation experiments (Allen, 1974).

RMSE is a frequently used measure of the differences between values predicted by a model or an estimator and the values observed. The RMSE represents the square root of the second sample moment of the differences between predicted values and observed values or the quadratic mean of these differences (Hyndman and Koehler, 2006).

Mean Absolute Error (MAE) is a measurement used to measure the average absolute difference between predicted and true values in regression problems. MAE can measure the average error between predicted and true values. The smaller the value of MAE, the smaller the average difference between predicted and true values, indicating a higher accuracy of prediction (Tropsha et al., 2003).

The Concordance Correlation Coefficient (CCC) was developed as a measure for the correlation between two sets of data, for instance a gold standard and a second reading (Sheikhpour et al., 2017).

The external predictivity of QSAR models is commonly described by employing validation metrics (Roy and Roy, 2008), such as *R* ^2^ based metrics, namely, Q^2^_F1_ and Q^2^_F2_ (Zhou and Sun, 1999; Golbraikh and Tropsha, 2002).

### 2.4 Linear model by HM

After generating molecular descriptors, HM in CODESSA software was used to accomplish the pre-selection of the descriptors and build the linear model (Si et al., 2021).

HM in CODESSA software has the advantages of high efficiency and no limitation to the size of the data set. After calculating all molecular descriptors, preprocessing program eliminates 3 types of descriptors that cannot be used: 1) descriptors that not all compounds have; 2) descriptors that have a small variation in magnitude for all structures; 3) two collinear descriptors with the correlation coefficient greater than 0.8 (Wang Y. et al., 2020). The number of remaining available molecular descriptors is marked N. The steps of HM can refer to literature (Katritzky et al., 1995).

The HM performs descriptors pre-selection by the following criteria (Katritzky et al., 2023): 1) Fisher F-criteria must be greater than 1.0; 2) *R* ^2^ value should be higher than a specified threshold; 3) Student’s t criterion must exceed a defined value; 4) duplicate descriptors should have a squared intercorrelation coefficient below a predetermined level, and the descriptor with a higher *R* ^2^ value relative to the property is retained. The remaining descriptors are then arranged in descending order based on their correlation coefficients. Any significant 2 parameter correlation identified by the F-criteria is further expanded recursively into an n parameter correlation until the normalized F-criteria is no longer higher than the initial value. The top N correlations which are determined by both *R* ^2^ and the F-criterion are then saved.

HM attempts to build a series of linear regression models with 1 to N molecular descriptors and find the optimal one. Molecular descriptors determined by HM model serve as independent variables of the non-linear models for the next step (Gao et al., 2022)^.^

### 2.5 Non-linear model by GEP

Considering that the relationship among the factors affecting IC_50_ of PARP1 inhibitors is complex and usually non-linear, one non-linear model was established by GEP to predict IC_50_ values of the compounds respectively.

As a new algorithm based on genetic algorithm (GA) and genetic planning (GP), GEP was proposed by Ferreira in 2001 (Ferreira, 2001). More details about GEP algorithm can refer to literature (Zhou and Sun, 1999). And the steps of GEP can refer to literature (Ferreira, 2001; Liu et al., 2004).

**FIGURE 1:**
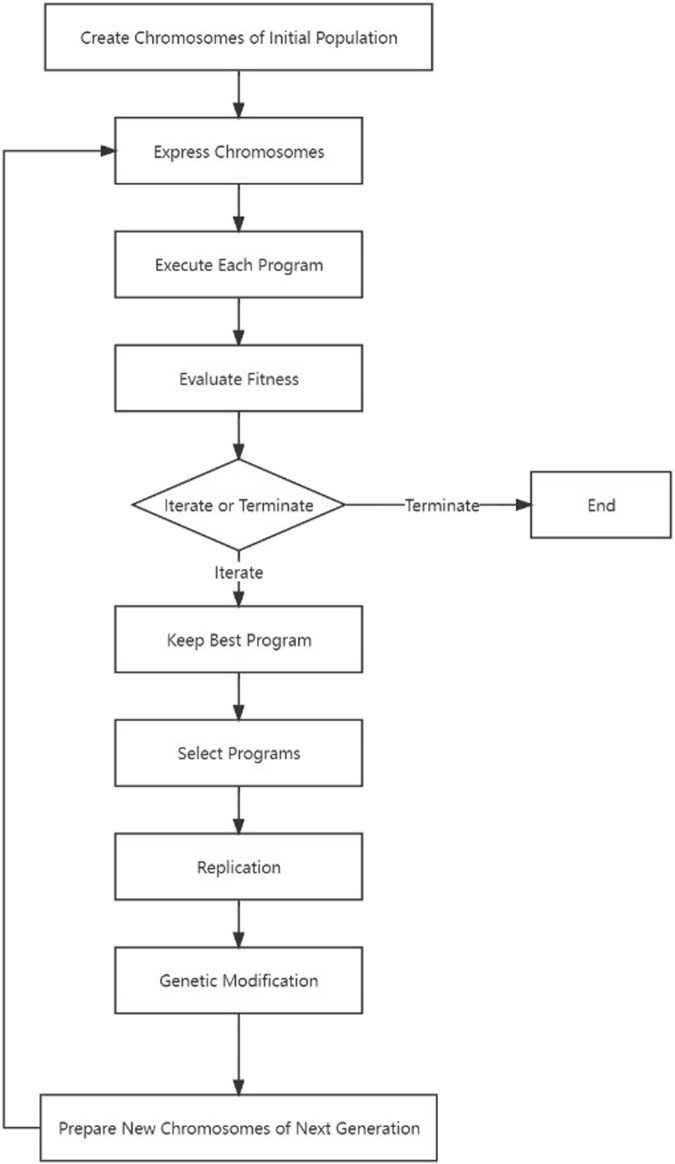
The process of gene expression programming.

The following schematic Figure 1 depicts the essential process of gene expression programming. Initially, a certain number of individuals (known as the initial population) have their chromosomes generated randomly. Subsequently, these chromosomes are expressed into syntactically correct programs, and each individual’s fitness is evaluated against a set of fitness cases, also referred to as the selection environment (Ferreira, 2001). Based on their fitness, individuals are selected and modified for reproducing offspring with new traits. These newly individuals undergo the same developmental process, including genome expression, evaluation within the selection environment, selection, and modified reproduction (Ferreira, 2006). The process is repeated for a specific number of generations or until a satisfactory solution is obtained.

GEP is widely used in science and Ferreira developed Automatic Problem Solver (APS) (Gepsoft, 2023), a commercial software integrated with GEP algorithm. The software can encode the appropriate molecular descriptors which are selected by HM and most related to inhibitor activity, and establish a non-linear model to predict IC_50_ values of FTPDDs.

### 2.6 Non-linear model by RF

Random forest regression is an ensemble learning algorithm that performs regression tasks by constructing multiple decision trees and integrating their prediction results. In random forest, each decision tree is trained on randomly selected subsets of the data, reducing the risk of overfitting. The final regression result is obtained by averaging or weighted averaging the predictions of the individual trees.

The algorithm works by randomly selecting samples from the training set to form subsets, increasing the diversity of the models. At each node of each decision tree, only a portion of randomly selected features are considered, improving the robustness of the models. Decision trees are built using recursive partitioning based on the least impurity. In random forest, the optimal splitting points of each tree are determined by calculating their Gini coefficients which are the measurements of the purity of a classification model.

The value range for the Gini coefficient is from 0 to 1. A larger value indicates a higher purity of the model. The classifier for random forest is the CART tree. The Gini coefficient of the CART tree can be represented by Eq. 1.

$$Gini\left(p\right)=2p\left(1-p\right)$$ (1)

When traversing each segmentation point of each feature, it is assumed that using the feature A = a. The set D is divided into two parts, namely, D1 (sample set that satisfies A = a) and D2 (sample set that does not satisfy A = a). The Gini coefficient of D under this feature is Eq. 2.

$$Gini\left(D,A\right)=\frac{\left|D_{1}\right|}{\left|D\right|}Gini\left(D_{1}\right)+\frac{\left|D_{2}\right|}{\left|D\right|}Gini\left(D_{2}\right)$$ (2)

Random forest regression has several advantages such as handling high-dimensional and large-scale datasets, having good generalization performance to avoid overfitting, and handling missing and abnormal values. Additionally, it has strong fitting ability for data with nonlinear relationships. The non-linear model established through random forest can effectively predict the IC_50_ value of FTPDDs.

### 2.7 Non-linear models by SVR

In order to build the model with stronger prediction ability, three non-linear models were established based on SVR with single, double, and triple kernel function respectively.

SVR is an important application branch of support vector machine (SVM). SVM, proposed by Vladimir N. Vapnik and Alexey Ya. Chervonenkis in 1964, is a generalized linear classifier (GLC) that classifies the data into binary categories under supervised learning. When SVM is applied in the field of regression analysis, it is often called SVR (Wang Y. et al., 2020). Unlike SVM which maximizes the distance from the hyperplane to the points closest to it, SVR minimizes the total deviation from all sample points to the hyperplane (Du and Wu, 2003).

A significant advantage of SVR is its simplicity in mathematical calculations, as it transforms nonlinear problems in the input space into linear problems in a high-dimensional feature space. Another advantage is that SVR can utilize probability rules to train multiple classifiers on different types of data, thus enhancing prediction accuracy by measuring the classification confidence. Compared to other regression techniques, SVR demonstrates lower computational complexity.

Some parameters of support vector machines play an important role in model training. The penalty constant (C) is a regularization parameter that controls the degree of punishment for misclassified samples. A larger C value can lead to stricter classification, which may lead to the model overfitting the training data. A smaller C value allows for more classification errors, which may lead to better generalization ability.

Slack variable, which introduces tolerance that allows some samples to be at the boundary of classification errors or intervals. These parameters are usually used in conjunction with C to adjust the degree of cosmetic error.

For certain kernel functions, such as polynomial kernels and RBF kernels, there can also be additional parameters, such as the order of the polynomial, the bandwidth of the Gaussian kernel, etc. These parameters need to be adjusted according to the characteristics of the data. For imbalanced datasets, the importance of different categories can be balanced by adjusting their weights. This is very useful when dealing with imbalanced data.

The basic idea of SVR is to use a predetermined non-linear constructor to map the input vector to the high-dimensional feature space and regress in the mapped space. To avoid complex mapping operations in high-dimensional feature space, kernel function is used to realize inner product operation in the original space (Fang and Zhao, 2013).

Referring to literature (Deng and Tian, 2004), the primal problem of standard ε-SVR is as follows.

$$\begin{array}{l} \min_{\omega \in R;\xi_{i},\xi_{i}^{*},b\in R}\frac{1}{2}{\left\|\omega \right\|}^{2}+\frac{C}{l}\sum_{i=1}^{l}\left(\xi_{i}+\xi_{i}^{*}\right),\\ s\cdot t\cdot \left(\left(\omega \cdot x_{i}\right)+b\right)-y_{i}\leq \varepsilon +\xi_{i},\\ y_{i}-\left(\left(\omega \cdot x_{i}\right)+b\right)\leq \varepsilon +\xi_{i}^{*},\\ \xi_{i}^{*},\xi_{i}\geq 0,i=1,2,\ldots ,l. \end{array}$$ (3)

In Eq. 3, ${\xi_{i}}^{\left(*\right)}$ means ${\left(\xi_{1},{\xi_{1}}^{*},\ldots ,\xi_{k},{\xi_{k}}^{*}\right)}^{T}$ , which is the slack variable. b is the offset. ε is the maximum error allowed in regression. C is the penalty constant. The problem in Eq. 3 can be transformed into its dual problem:

$$\begin{array}{l} \min_{\alpha^{\left(*\right)}\in R^{2l}}\frac{1}{2}\sum_{i,j=1}^{l}\left(\left(\alpha_{i}^{*}-\alpha_{i}\right)\right.\left(\alpha_{j}^{*}-\alpha_{j}\right)K\left(x_{i,}x_{j}\right))+\\ \varepsilon \sum_{i=1}^{l}\left(\alpha_{i}^{*}+\alpha_{i}\right)-\sum_{i=1}^{l}y_{i}\left(\alpha_{i}^{*}+\alpha_{i}\right),\\ s\cdot t\cdot \sum_{i=1}^{l}\left(\alpha_{i}-\alpha_{i}^{*}\right)=0,\\ 0\leq \alpha_{i},\alpha_{i}^{*}\leq \frac{C}{l},i=1,2\ldots ,l. \end{array}$$ (4)

In this Eq. 4, $\alpha^{\left(*\right)}$ means ${\left(\alpha_{1},{\alpha_{1}}^{*},\ldots ,\alpha_{l},{\alpha_{l}}^{*}\right)}^{T}$ which is Lagrange multiplier and $K\left(x_{i},y_{i}\right)$ is kernel function. By solving this dual problem, the answer to the primal problem and the final regression decision function are obtained.

According to the quadratic programming method in the optimization theory, the parameters $\alpha_{1}$ and $\alpha_{1}^{*}$ can be obtained in solving the dual problem. The parameter b can be gotten by using the Karush-Kuhn-Tucker (KTT) condition. In this way, the expression of the fitting function on the sample set $f\left(x\right)$ can be constructed. Its form is given in Eq. 5. The coefficient $\left(\alpha_{i}-\alpha_{i}^{*}\right)\neq 0$ is the support vector.

$$f\left(x\right)=\sum_{i=1}^{l}\left(\alpha_{i},-\alpha_{i}^{*}\right)*K\left(x_{i},x_{j}\right)+b$$ (5)

### 2.8 Kernel function of SVR

Kernel function has a great influence on the fitting effect of the SVR model. As mentioned in the part of establishing non-linear models by SVR, the job of the kernel function that realizes the inner product operation in the original space is to simplify calculation in high-dimensional space. Kernel function maps each sample point to an infinite-dimensional feature space to make the linearly inseparable data linearly separable (Wang and Chen, 2014). The popularly used kernel functions include linear kernel function, RBF kernel function, polynomial kernel function, and sigmoid kernel function (Mo et al., 2020). The first three kernel functions have been widely applied when establishing models by SVR and their introduction is as follows (Sheikhpour et al., 2018).

Linear kernel function is the simplest kernel function, which only calculates the inner product of two feature vectors. The accuracy of linear kernel function is not high (Mo et al., 2020) but it can be used to find which feature vectors are important with little computation. The form of linear kernel function is as follows.

$$K_{L}\left(x,x_{i}\right)=\left(x^{T}x_{i}\right)$$ (6)

RBF kernel function, a certain kind of scalar function that’s radially symmetric, is the most widely used kernel function. RBF kernel function has a strong local learning ability, which means it can well characterize the local property of the sample, but its generalization ability is not good enough (Fang and Zhao, 2013; Ding et al., 2021). The form of RBF kernel function is given in Eq. 7, where the constant σ is the kernel radius of RBF kernel function.

$$K_{RBF}\left(x,x_{i}\right)=\exp\left\{-\frac{{\left\|x-x_{i}\right\|}^{2}}{2\sigma^{2}}\right\}$$ (7)

The polynomial kernel function is a commonly used kernel function. The generalization ability of polynomial kernel function is strong, but its local learning ability is not good. The form of polynomial kernel function is given in Eq. 8, where the constant q is the order of polynomial kernel function.

$$K_{Poly}\left(x,x_{i}\right)={\left[\left(x^{T}x_{i}\right)\right]}^{q}$$ (8)

The process of establishing three models of SVR with different number of kernel functions is as follows.

First, SVR with single kernel function was used to establish the regression model. RBF kernel function is usually selected as the single kernel function due to its good local learning ability. The form of single kernel function is as follows.

$$K\sin gle=K_{RBF}$$ (9)

In order to obtain the kernel function that enhances the learning and generalization ability of the model by SVR, Smits et al. proposed the multiple kernel function (Fang and Zhao, 2013). There are many combinations of kernel function, but the obtained multiple kernel function must satisfy Mercer’s Theorem (Smits and Jordaan, 2002).

According to the closure characteristic of kernel functions, the derivation principle of mixed kernel functions can be strictly proven. Take $\left\{X1,X2,\cdot \cdot \cdot ,Xm\right\}\subseteq X\subseteq R^{n}$ , the Gram matrices corresponding to $\left\{X1,X2,\cdot \cdot \cdot ,Xm\right\}K_{1}$ and $K_{2}$ are both positive definite matrices. If $c\subseteq R^{n}$ is chosen, then *c′(* α *K*_1_ + β *K*_2_ *)c = c′* α *K*_1_ *c* + $c^{\prime }\beta$ *K*_2_ *c ≥* 0 indicating that the Gram matrices corresponding to $\alpha K_{1}+\beta K_{2}$ are all positive definite. From this, it can be concluded that $\alpha K_{1}+\beta K_{2},\alpha ,\beta \geq 0$ is kernel function. In addition, by limiting α+β=1 , a stationary mixed kernel function can be constructed. Because the regression ability and generalization ability of SVR model are a pair of mutually balanced factors, the SVR model obtained by mixed kernel functions with different properties in a certain proportion can balance the performance of both aspects.

Considering that the generalization ability of polynomial kernel function is strong, RBF kernel function and polynomial kernel function were integrated as double kernel function to establish the SVR model with enhanced generalization ability (Yang et al., 2023). The form of double kernel function is given in Eq. 10, where the value of the variable a ranges from 0 to 1.

$$K_{dual}=a*K_{RBF}+\left(1-a\right)*K_{poly}$$ (10)

Considering linear kernel function can capture key vectors at the cost of little computation and therefore help to enhance the regression effects of the model, linear kernel function was added to the previous double kernel function, which formed triple kernel function to establish the regression model with better learning and generalization ability again. The form of triple kernel function is given in Eq. 11, where variables a and b are positive coefficients and the sum of a and b is less than or equal to 1.

$$K_{triple}=a*K_{RBF}+b*K_{poly}+\left(1-a-b\right)*K_{L}$$ (11)

Therefore, three models based on SVR with different numbers of kernel functions were constructed to predict the IC_50_ values of compounds.

### 2.9 SVR model optimized by particle swarm optimization

The values of parameters when establishing SVR model have a great influence on the learning and generalization ability of the model. In the process of constructing the model by SVR with triple kernel function, six parameters need to be optimized: the insensitive parameter ε , the penalty factor C , the kernel radius of RBF kernel function σ , the coefficient of RBF kernel function a , the order of polynomial kernel function q , and the coefficient of polynomial kernel function b . And in the process of establishing the models by SVR with single and double kernel function, the first three and five parameters need to be optimized respectively. Their searching ranges are as follows. $\varepsilon \in \left[0,0.8\right]$ , $C\in \left[0.001,200\right]$ , $\sigma \in \left[0.001,5\right]$ , $a\in \left[0,1\right]$ , $q\in \left\{1,2,3\right\}$ , $b\in \left[0,1-a\right]$ .

However, the optimization of parameters when building model by SVR with single kernel function has already become a research difficulty. As for the model by SVR with multiple kernel function, with the number of parameters increasing, it is more difficult to optimize the parameters. Considering that traditional parameter-searching methods including the grid search method and the random search method are inefficient, particle swarm optimization (PSO) algorithm was used to optimize parameters in the process of establishing three models by SVR.

As a kind of bionic optimization algorithm, PSO was proposed by social psychologist Kennedy and electrical engineer Eberhart in 1995. The idea of PSO is based on birds swarm finding the optimal destination by sharing collective information. PSO algorithm takes random values in high-dimensional space to initialize the position and velocity information of particles and update the information by self and group learning of particles. In PSO algorithm, particles only transmit the optimal information in the process of iterative process, so the PSO has the advantages of fast search speed and convergence rate (Wang, 2022).

PSO adopts the velocity-position model, in which the forms of position and velocity of particle i in D-dimensional solution space are as follows.

$$Xi=\left(x_{i1},x_{i2},x_{i3},\ldots ,x_{iD}\right)$$ (12)

$$Vi=\left(v_{i1},v_{i2},v_{i3},\ldots ,v_{iD}\right)$$ (13)

To characterize which particle is in the best position among all particles, RMSE is used to evaluate the fitness of each particle. The smaller RMSE means better position and fitness. The best position of particle i is recorded as $p_{ibest}$ and the global best position is recorded as $g_{best}$ . In each iteration, the particle tracks $p_{ibest}$ , $g_{best}$ and its previous state to adjust the position and velocity at the current time. The iterative equations are as follows.

$$v_{i}\left(k+1\right)=w*v_{i}\left(k\right)+c_{1}*rand\left(\;\right)*\left(p_{ibest}-x_{i}\left(k\right)\right)+c_{2}*rand\left(\;\right)*\left(g_{best}-x_{i}\left(k\right)\right)$$ (14)

$$X_{i}\left(k+1\right)=X_{i}\left(k\right)+V_{i}\left(k+1\right)$$ (15)

In the Eq. 15, $V_{i}\left(k\right)$ , $V_{i}\left(k+1\right)$ , $X_{i}\left(k\right)$ , $X_{i}\left(k+1\right)$ are the velocity and position of the particle i at the current time and the next time respectively; $rand\left(\;\right)$ is a random number in range $\left[0,1\right]$ . $c_{1}$ and $c_{2}$ are learning factors which are usually set to 2. ω is the weight factor and the value of ω automatically decreases with the iteration of the algorithm to accelerate the convergence rate. It is generally defined as:

$$\omega =\omega_{\min}+\left(iter_{\max}-iter\right)*\left(\omega_{\max}-\omega_{\min}\right)/iter_{\max}$$ (16)

$\omega_{\max}$ , $\omega_{\min}$ are the maximal and minimal weight factors respectively, iter is the current number of iterations, and $iter_{\max}$ is the maximum number of iterations.

Referring to literature (Xiong and Xu, 2006), The steps of parameter optimization by PSO are as follows.

Step 1 Initialize parameters of PSO including population size and the maximal and minimal weight factors $\omega_{\max}$ and $\omega_{\min}$ . Set the maximum number of iterators $iter_{\max}$ .

Step 2 Each particle is randomly assigned a set of position information $X_{\left(i,0\right)}$ and velocity information $V_{\left(i,0\right)}$ . Set the individual best position $p_{ibest}$ of each particle to its current position $X_{\left(i,0\right)}$ .

Step 3 RMSE values of each particle are calculated to evaluate fitness. The initial global best position $g_{best}$ is set to the position of the particle with the best fitness.

Step 4 Update the position and velocity of each particle by iterative calculation according to Eqs 14–16 and calculate the RMSE of each particle to evaluate fitness.

Step 5 Compare the fitness of each particle with the fitness of its $p_{ibest}$ . If the fitness is better than that of $p_{ibest}$ , $p_{ibest}$ is updated to the current position, otherwise the original value of $p_{ibest}$ remains unchanged.

Step 6 Compare the updated $p_{ibest}$ of each particle with $g_{best}$ . If $p_{ibest}$ is better than $g_{best}$ , $g_{best}$ is updated to $p_{ibest}$ , otherwise the original value of $g_{best}$ remains unchanged.

Step 7 Judge whether the termination conditions are met. If the maximum number of iterations is reached or $g_{best}$ is not changed, terminate the iteration, otherwise return to step 4.

### 2.10 Property prediction and molecular docking

It is essential for newly designed molecules not only to exhibit efficacy against the target but also to possess favorable physical and chemical properties. Property explorer is a complimentary tool that predicts physical, chemical, and toxicological properties of molecules to aid in designing active drug compounds (Song et al., 2017). This tool enables researchers to construct new molecular structures and analyze their attribute values automatically. It provides valuable information on properties such as molecular weight, Partition coefficient (LogP), water solubility, topological polar surface area (TPSA), drug similarity, toxicity assessment, overall drug score, etc.

Macromolecular docking has become a key step in the drug development process (Chen et al., 2022a), facilitating the identification of potential therapeutic molecules and predicting ligand-target interactions at the molecular level (Chen et al., 2022b). To explore these interactions between the new PARP inhibitor and PARP1 at the binding site, the Sybyl-X 2.1 software package was employed (Li et al., 2012) This software allowed users to generate potential interactions between the molecules and proteins, as well as exploring the expected binding sites for molecular fitting.

The macromolecular docking technique was utilized to investigate the potential interaction between the new PARP inhibitor and the binding site of PARP1. Firstly, the chemical structure was imported into Sybyl-X software for calculation and optimization with parameters “max interactions” and “max display” as 1000 and 0.01, respectively. The molecules were then assigned Gasteiger-Hückel charges and minimized using the Tripos force field until convergence reached 0.05 kcal/mol/Å (Song et al., 2023).

Subsequently, the protein was imported into Sybyl-X software for hydrogenation, charging, and optimization. Unnecessary ligands and water molecules were removed to enable proper binding with the protein targets.

Finally, the docking results were imported into PyMol software for image optimization, and the amino acid residues and hydrogen bonds were labeled by same software.

## 3 Results and discussion

### 3.1 Lg (IC_50_) of FTPDDs

### 3.2 Results of HM

**FIGURE 2:**
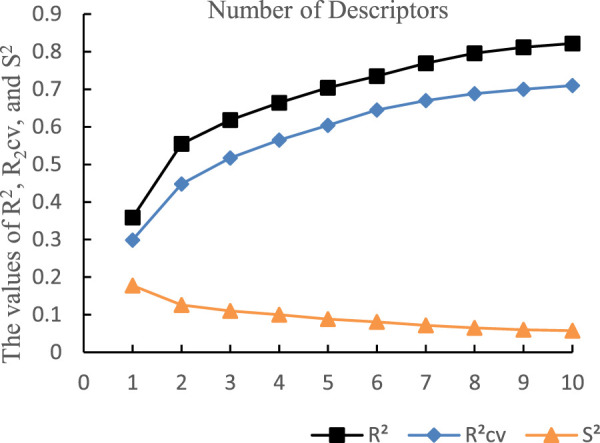
Influences of the number of descriptors on R2, R2cv, and S2 of all compounds.

540 molecular descriptors were calculated in CODESSA software. To select the molecular descriptors most related to PARP1 inhibitors, a series of linear models with the increasing number of molecular descriptors were established. Figure 2 shows the influences of the number of descriptors on *R* ^2^, R^2^_cv_, and the standard deviation (S) of all compounds.

As shown in Figure 2, the values of *R* ^2^ and R^2^_cv_ of all compounds were rising with the increasing number of molecular descriptors. However, when the number of descriptors reached eight, adding another descriptor did not significantly improve the statistics of the model, so the eight-parameter model can be regarded as the optimal one. The eight selected molecular descriptors and their physical-chemical meanings are shown in Table 5, and their *R* ^2^ matrix is shown in Table 6. The 8-parameter model is discussed in details as follows.

**TABLE 5:** The selected descriptors and their physical-chemical meanings and coefficient.

Symbol	Physical-chemical meaning	Coefficient
MENANNB	Min e-n attraction for a N-N bond	0.54386
MEEROA	Min e-e repulsion for a O atom	−0.19081
MRECCB	Max resonance energy for a C-C bond	0.45861
MBCM	Max bonding contribution of a MO	7.2924
ANRINA	Avg nucleoph. react. index for a N atom	−112.51
KA (O3)	Kier shape index (order 3)	−0.58196
HDH/T (QCP)	HA dependent HDCA-2/TMSA [Quantum-Chemical PC]	−192.22
MRECHB	Max resonance energy for a C-H bond	−7.1692

**TABLE 6:** *R* ^2^ matrix of the eight descriptors.

Descriptor	MENANNB	MEEROA	MRECCB	MBCM	ANRINA	KA (O3)	HDH/T(QCP)	MRECHB
MENANNB	1.0000							
MEEROA	0.0724	1.0000						
MRECCB	0.0479	−0.0651	1.0000					
MBCM	0.5892	0.1741	−0.0281	1.0000				
ANRINA	−0.1022	−0.1146	−0.3241	0.1031	1.0000			
KA (O3)	0.5518	−0.1184	0.2525	0.1826	−0.5942	1.0000		
HDH/T (QCP)	−0.6837	−0.0693	0.0308	−0.5378	−0.0850	−0.5761	1.0000	
MRECHB	0.3572	0.0891	0.1132	0.4526	0.0481	−0.0158	−0.1233	1.0000

**FIGURE 3:**
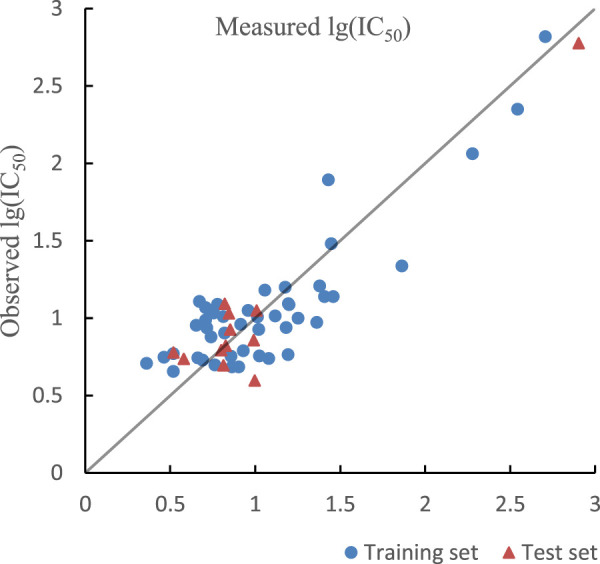
Plot of measured and predicted lg (IC50) by HM.

*R* ^2^ of training set and test set in HM model were 0.7550, 0.9014 and their RMSE were 0.2327, 0.2378. The optimal prediction results by HM model are shown in Figure 3. In addition, R^2^_cv_ of training set and test set in HM model are 0.7773 and 0.6798.

### 3.3 Results of GEP

**FIGURE 4:**
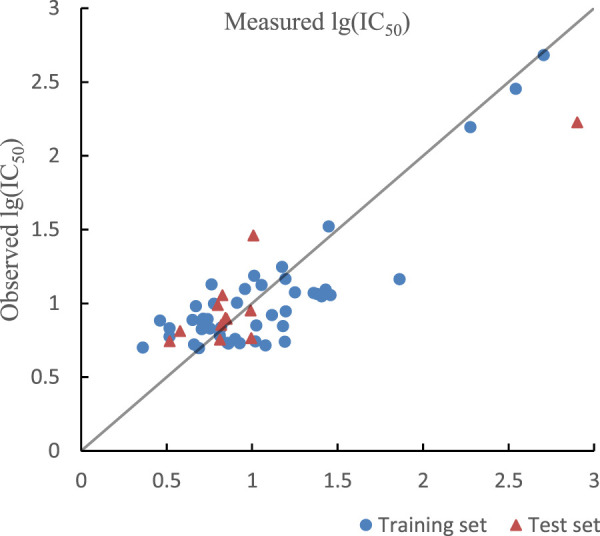
Plot of measured and predicted lg (IC50) by GEP.

The same eight descriptors were imported into APS software to search the ideal model by GEP. Then the optimal model was obtained in the 199th generation. *R* ^2^ of training set and test set in GEP model were 0.7395, 0.7818 and their RMSE were 0.2520 and 0.2768. R^2^_cv_ of training set and test set in GEP model were 0.7302 and 0.6998. The optimal prediction results by GEP model are shown in Figure 4. The mathematical equation of the non-linear model built by GEP is as follows.

$$\begin{array}{l} \mathrm{lg}\left(\text{IC}50\right)=floor\left(d_{5}\right)+\sec\left(d_{3}\right)+d5*d2+\tan\left(\log stic\left(\left(d0*d_{1}\right)*\left(-d_{1}\right)\right)\right)\\ {}*\mathrm{arccot}\left(d_{2}\right)+floor\left(\sin\left(\left(d_{3}-\arctan\left(d_{4}\right)\right)-\left(floor\left(d_{7}\right)+d_{1}\right)\right)\right) \end{array}$$ (17)

In Eq. 17, d0, d1, d2, d3, d4, d5, d6, and d7 represent MENANNB, MEEROA, MRECCB, MBCM, ANRINA, KA (O3), HDH/T (QCP), and MRECHB.

### 3.4 Results of RF

**FIGURE 5:**
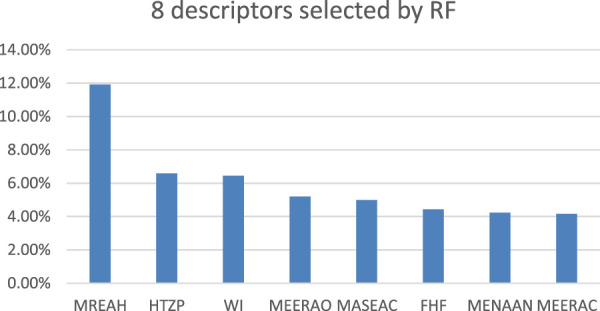
Eight descriptors selected by RF.

In order to verify the effectiveness of selecting descriptors through the HM method, RF was applied in the experiment for feature selection. Firstly, 540 unprocessed molecular descriptors belonging to 57 FTPDDs were exported from Codessa. Secondly, all descriptors containing default values were removed. Thirdly, according to the preprocessing steps of the heuristic algorithm, all descriptors with high collinearity and irrationality were also removed. Finally, RF was used to extract features from the remaining descriptors to select the most important 8 descriptors. Figure 5 shows the 8 descriptors selected by RF. The comparison between the descriptors selected by RF and HM was carried out to verify the effectiveness of the descriptors.

The 8 descriptors filtered out are Max resonance energy for a H-N bond (MREAH), HASA-2/TMSA [Zefirov’s PC] (HTZP), Wiener index (WI), Min e-e repulsion for a O atom (MEERAO), Max atomic state energy for a C atom (MASEAC), Final heat of formation (FHF), Min e-n attraction for a N-N bond (MENAAN), Min e-e repulsion for a C-H bond (MEERAC). Min e-e repetition for a O atom and Min e-n attraction for a N-N bond are the two most important descriptors selected through HM. Both descriptors were successfully screened by RF, demonstrating the effectiveness of the HM algorithm.

Furthermore,5-fold cross-validation was used to obtain an R^2^_cv_ of 0.87 for the training set and 0.86 for the test set. The MAE values for the training and testing sets are 0.1518 and 0.0821, respectively. In addition, the RMSE of four ring compounds and five ring compounds are 0.3078 and 0.3420, respectively, and their *R* ^2^ values are 0.7203 and 0.9002, respectively.

### 3.5 Results of SVR

The learning and generalization ability of SVR model depends on kernel function and the values of parameters. In the process of establishing models by SVR, there are always two parameters to be optimized: the insensitive parameter ε and the penalty factor. The analysis of the influences of the parameters to the SVR model (Fang and Zhao, 2013) is as follows. ε reflects the sensitivity of the model to the noise contained in the input vectors. The larger the ε is, the lower the fitting accuracy of the model is. C represents the tolerance of the model to the error. The greater the C is, the higher the fitting accuracy is, which causes more computation. In addition, if the model is established by SVR with multiple kernel function, the coefficients of kernel functions also need to be optimized. Considering the low efficiency of traditional parameter searching algorithms including the grid search method and the random research method when the number of parameters is increasing, PSO was employed to find the best combination of parameters. In PSO algorithm, only the optimal information is transmitted between particles, which avoids searching all combinations of parameters and accelerates convergence rate.

SVR with single kernel function is commonly used, in which RBF kernel function is usually selected. In Eq. 7, the kernel radius σ represents the mean square deviation of RBF function, that is, the width of RBF kernel function in the direction of independent variable. The smaller the σ is, the better the fitting performance of RBF kernel function is, which leads to poor generalization ability (Fang and Zhao, 2013). The optimal combination of parameters by PSO is as follows.

$$\left(\varepsilon ,C,\sigma \right)=\left(0.024,4.132,0.758\right)$$

**FIGURE 6:**
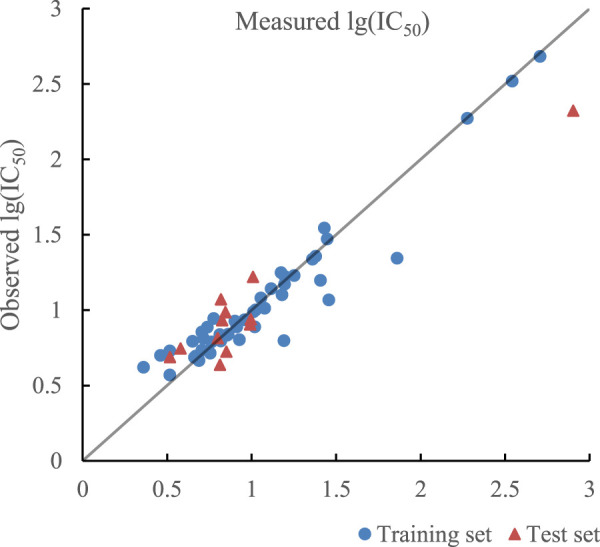
Plot of measured and predicted lg (IC50) by SVR with single kernel function.

The optimal prediction results of the model established by SVR with single kernel function are given in Figure 6. *R* ^2^ of training set and test set in the model were 0.9114 and 0.8599 and their RMSE were 0.0215 and 0.0491 respectively. In addition, R^2^_cv_ of training set and test set in the model were 0.8223 and 0.7815.

Results showed that *R* ^2^ of training set was about 0.06 more than that of test set in the model established by SVR with single kernel function, which meant the model had an unsatisfactory generalization ability. Therefore, polynomial kernel function was integrated with RBF kernel function to establish the model by SVR with double kernel function. Therefore, the coefficient of RBF kernel function a and the order of polynomial kernel function q also need to be optimized. The optimal combination of parameters by PSO is as follows.

$$\left(\varepsilon ,C,\sigma ,a,q\right)=\left(0.013,0.658,0.328,0.51,2\right)$$

**FIGURE 7:**
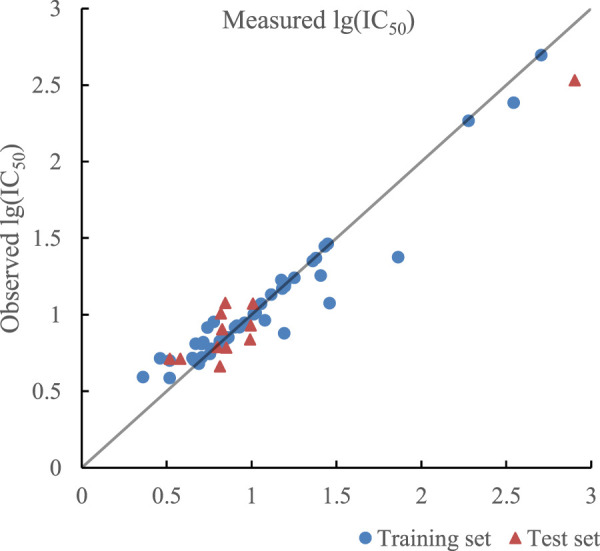
Plot of measured and predicted lg (IC50) by SVR with double kernel function.

The ratio of RBF kernel function to polynomial kernel function was 0.51:0.49, which meant RBF kernel function and polynomial kernel function play almost equal roles. The optimal prediction results of the model established by SVR with double kernel function are given in Figure 7. *R* ^2^ of training set and test set in the model were 0.9259 and 0.9175 and their RMSE were 0.0180 and 0.0289 respectively. In addition, R^2^_cv_ of training set and test set in the model were 0.8989 and 0.8712.

However, results showed that both R^2^_cv_ of training set and test set were less than 0.9 in the model established by SVR with double kernel function, which meant the learning and generalization ability of the model still needed to be improved. As mentioned before, linear kernel function can improve the regression ability of the model, so linear kernel function, polynomial kernel function, and RBF kernel function composed triple kernel function, which formed a novel SVR model with better learning and generalization ability. The optimal combination of parameters by PSO is as follows.

$$\left(\varepsilon ,C,\sigma ,a,q,b\right)=\left(0.024,1.123,0.332,0.31,2,0.58\right)$$

**FIGURE 8:**
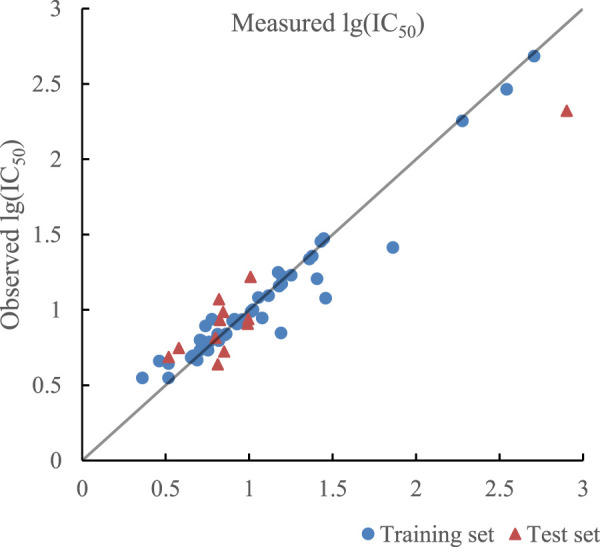
Plot of measured and predicted lg (IC50) by SVR with triple kernel function.

The ratio of RBF kernel function, polynomial kernel function, and linear kernel function was 0.31:0.58:0.11, which indicated that polynomial kernel function played the most important role in realizing inner product operation of kernel function. The optimal prediction results of SVR with triple kernel function are given in Figure 8. *R* ^2^ of training set and test set in the model were 0.9353 and 0.9348 and their RMSE were 0.0157 and 0.0228 respectively. In addition, R^2^_cv_ of training set and test set in the model were 0.9090 and 0.8971.

### 3.6 Design of new FTPDDs

Through the analysis of the molecular descriptors adopted in models, the structural factors that influence the IC_50_ values of the FTPDDs were identified. The non-standardized coefficients in table 2 represent the slope of the regression equation for each independent variable and indicate the magnitude of change in the dependent variable (IC_50_) with respect to each independent variable. These coefficients are not dependent on the unit of the independent variables and can reveal the influence of various factors on IC_50_ activity.

The descriptors included in the HM model provide valuable insights into the factors related to IC_50_ activity:

(1) “MENANNB” refers to the interaction force between the electrons and nucleus in the bond between two nitrogen atoms. Reducing this value leads to a significant reduction in IC_50_. (Clementi, 1980).
(2) “MEEROA” represents the minimum recurrence between electrons in an oxygen atom. As its coefficient in the HM model is negative, increasing the MEEROA value results in gradually decrease in the IC_50_ value. (Clementi, 1980).
(3) “MRECCB” reflects the maximum response energy of a C-C bond. In the HM model, it has a positive regression coefficient, meaning that smaller MRECCB value indicates stronger inhibitory ability of PARP. (Clementi, 1980).
(4) “MBCM” quantifies the contribution of each atomic orbital in a molecular orbital to the formation of a chemical bond. In the HM model, a positive regression coefficient suggests that smaller MBCM value leads to a lower IC_50_ value and higher activity. (Clementi, 1980).
(5) “ANRINA” describes the relative reactivity of nitrogen atoms as nuclear agents in chemical reactions. The negative coefficient in the HM model implies that increasing the ANRINA value leads to gradual decrease in the IC_50_ value. (Franke, 1984).
(6) “KA (O3)” reflects the connectivity and arrangement of atoms in a molecule. The negative regression coefficient indicates that larger KA (O3) value corresponds to a smaller IC_50_ value and higher activity. (Kier, 1985).
(7) “HDH/T (QCP)” describes hydrogen bond interactions on the molecular surface. A negative regression coefficient suggests that higher HDH/T (QCP) value leads to a smaller IC_50_ value and higher activity. (Clementi, 1980).
(8) “MRECGB” refers to the context of a molecular orbital (MO) particle in bonding in a chemical molecule or specifications. In the HM model, the negative regression coefficient indicates that larger MRECGB value results in a smaller IC_50_ value and higher activity. (Clementi, 1980).

In summary, the interpretation of the HM model and molecular descriptors has revealed several factors affecting inhibitory activity. To design new compounds, it is beneficial to reduce polar interactions between molecular atoms and alter the characteristics of different charge distributions of N atoms.

**FIGURE 9:**
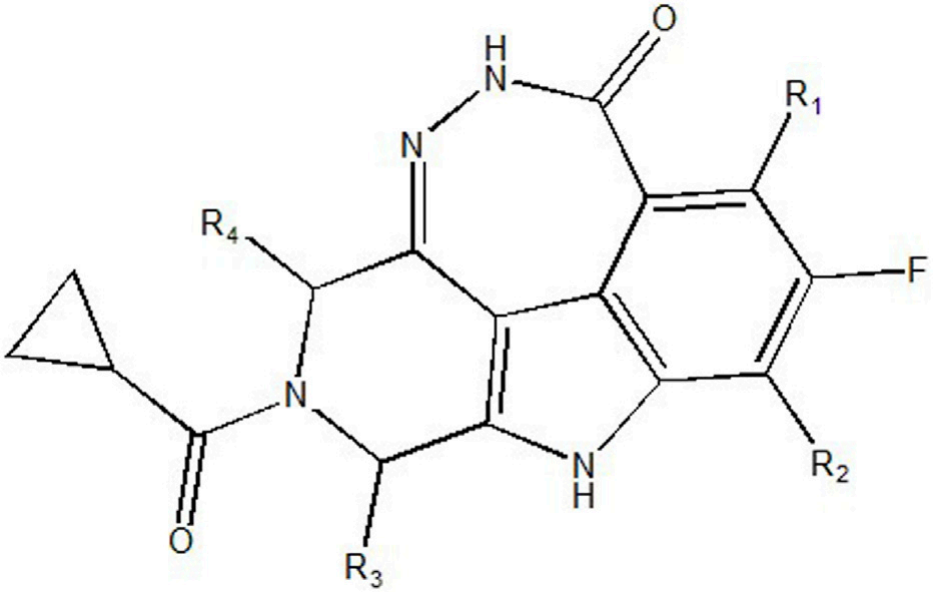
The design strategy mainly focused on the R region of compound 44.

To obtain an ideal inhibitor structure, the structural composition of compound 44 which is the most effective compound in the literature can be modified based on these factors. Molecular structure adjustments can focus on the R region shown in Figure 9. Specifically, changes in the benzene ring with its 6 C atoms may be favorable for achieving the desired distribution of different charges.

Some chemical functional groups were incorporated into positions R_1_ to R_4_, utilizing a random combination approach to minimize polar interactions between atoms. These functional groups include halogen, hydroxyl, carboxyl, aldehyde, hydrocarbon, as well as various forms of carbon and nitrogen atoms.

Through continuous and purposeful adjustments and combinations, a set of 126 molecules was designed based on analysis of descriptors in the HM model. Subsequently, the physical and chemical parameters of the newly designed molecules were calculated using CODESSA software. By inputting these parameters into the HM model, the IC_50_ value of each molecule was predicted. If the predicted IC_50_ value was lower than that of compound 44, the corresponding molecule would be selected for further analysis and macromolecular docking study in the Property explorer applet. Table 7 shows the predicted IC50 by HM and Docking total score of new FTPDDs.

**TABLE 7:** Predicted IC50 by HM and Docking total score of new FTPDDs.

No.	FTPDDs	Predicted IC_50_	Total score
44	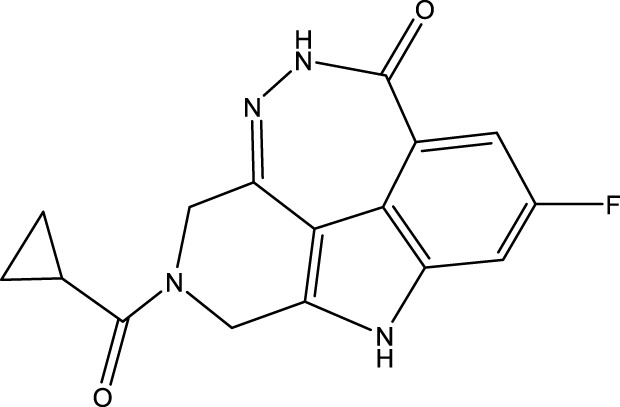	0.7052	6.3154
44a	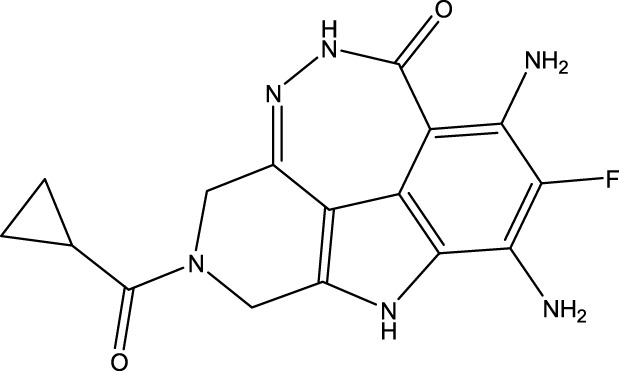	0.5546	7.7065
44b	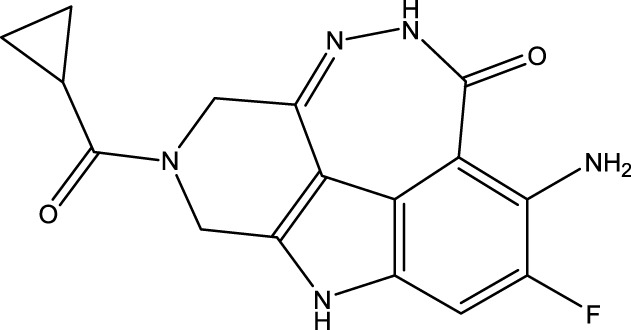	0.5516	6.7748
44c	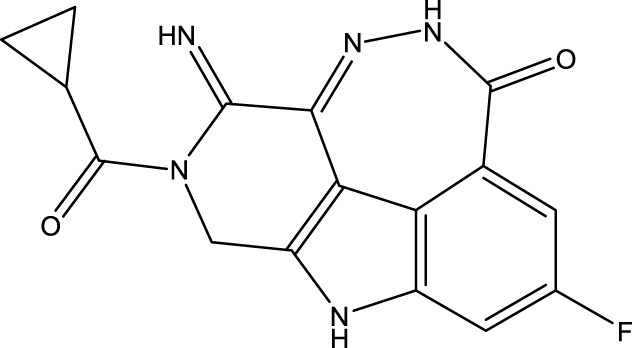	0.5036	6.8673
44d	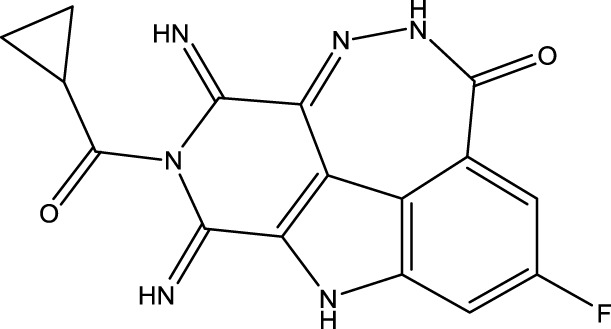	0.4402	6.4417
44e	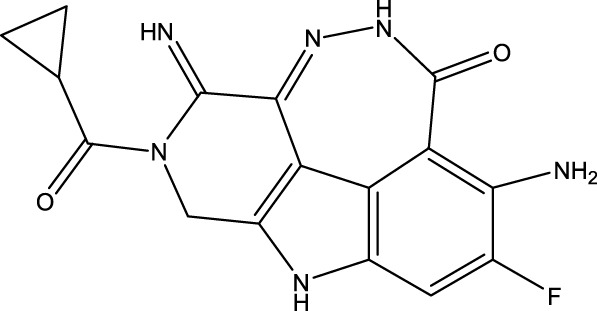	0.3561	6.3002
44f	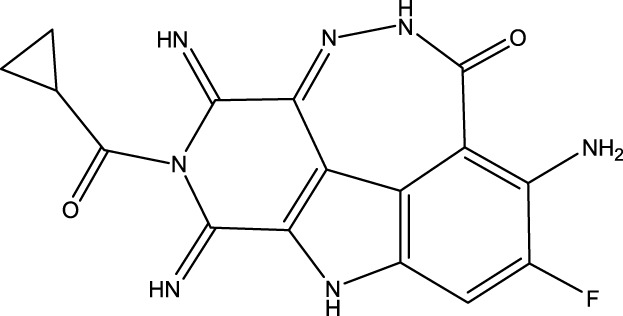	0.3951	6.8984

As a result of the analysis, the predicted IC_50_ values of six new compounds indicate that these compounds have preferable properties are worth further research. The predicted IC_50_ values of all these compounds were lower than the predicted values obtained in HM training for compound 44.

### 3.7 Property prediction of new FTPDDs

In order to predict properties of the new compound, the Property explorer applet (PEA) was applied in the experiment. This applet provides real-time predictions of physico-chemical properties and identification of potential toxicity risks for any chemical structure drawn. It can evaluate many properties of compounds, including partition coefficient, water solubility, topological polar surface area (TPSA), molecular weight, etc. Table 8 shows the predicted IC_50_ by HM and properties by PEA of newly designed compounds.

**TABLE 8:** Predicted IC_50_ by HM and properties by PEA of newly designed compounds.

No.	Pre.IC_50_	Toxicity	Logp	Solubility	Mol weight	TPSA	Drug likeness	Drug score
44	0.7052	Medium	1.75	−4.13	326.0	77.56	6.72	0.63
44a	0.5546	Medium	−0.33	−4.17	342.0	129.6	6.23	0.63
44b	0.5516	Medium	−0.35	−4.09	327.0	103.5	6.32	0.64
44c	0.5036	Medium	−0.06	−3.18	325.0	101.4	5.99	0.70
44d	0.4402	Medium	−0.17	−2.49	338.0	126.2	5.05	0.72
44e	0.3561	Medium	−0.61	−3.25	340.0	127.4	5.94	0.69
44f	0.3951	Medium	−0.84	−2.56	353.0	151.2	4.99	0.71

The partition coefficient, abbreviated P, is defined as a particular ratio of the concentrations of a solute between the two solvents and the logP is the logarithm of the ratio. The LogP value represents the logarithm of the partition coefficient between n-octanol and water, which is a standard measure of a compound’s hydrophilicity. It has been established that the LogP value of compounds with good potential absorption should not exceed 5.0 (Kwon, 2001).

Water solubility significantly influences the intestinal absorption and cellular distribution characteristics of compounds. Higher solubility means better absorption. The goal of drug design is to obtain compounds with higher water solubility.

TPSA is the sum of all topological polar regions on the molecular surface and is closely related to various bioavailability-related characteristics, such as permeability through the Blood–brain barrier (Ertl et al., 2000).

Molecular weight plays a role in the biological activity of compounds. Lower molecular weight compounds are more easily absorbed and distributed.

Drug similarity is utilized in new drug design to evaluate the “similarity” of the compound with factors such as bioavailability (Smith, 2011).

### 3.8 Molecular docking of new FTPDDs

**FIGURE 10:**
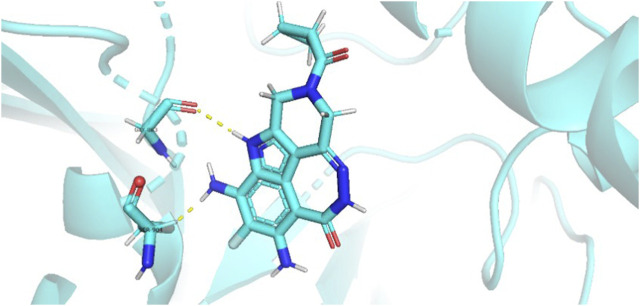
Docking assay of compound 44a with PARP related target (PDB ID: 7CMW).

During the lunar docking experiments, the newly designed compounds were employed as ligands to dock with PARP (pdb code 7CMW). Remarkably, compound 44a exhibited the most favorable performance in the macromolecular docking, achieving remarkable total score of 7.7065 which significantly surpassing that of compound 44. The detailed binding mode of compound 44a is presented in Figure 10, illustrating the formation of two crucial hydrogen bonds with specific residues.

Based on the docking conformation of compound 44a, the N atom establishes a hydrogen bond with the residue GLY-863, which aligns with the binding pattern observed in compound 44. Additionally, the nitrogen atoms from the newly incorporated structural component also form hydrogen bonds with SER-904. The strong bond reaction observed between compound 44a and PARP suggests that it could potentially serve as a promising candidate inhibitor for this protease.

Molecular docking requires the preparation of ligands and protein structures. Maestro is required for Protein Preparation. Firstly, energy minimization is performed, and then the receptor is isolated from the protein. Minimize the energy of the generated compound in Chem3D using MM2, align the processed compound as a ligand in PyMol, and finally calculate the RMSD between the generated compound and the existing ligand (Wang and Zhang, 2023). Table 9 shows RMSD values between the selected docking pose of 7 cmw and the experimental X-ray structure.

**TABLE 9:** RMSD values between the selected docking pose of 7 cmw and the experimental X-ray structure.

No.	FTPDDs	RMSD (A˚)
44	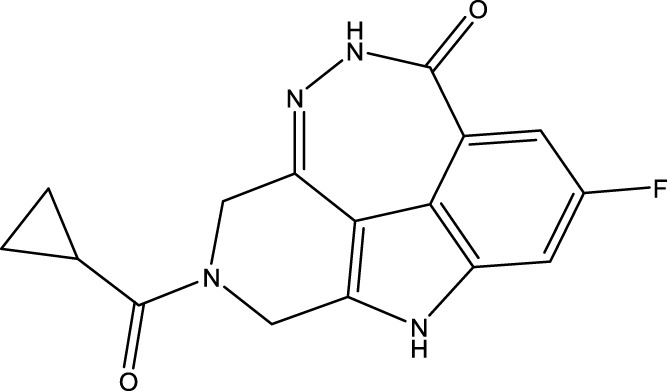	2.802
44a	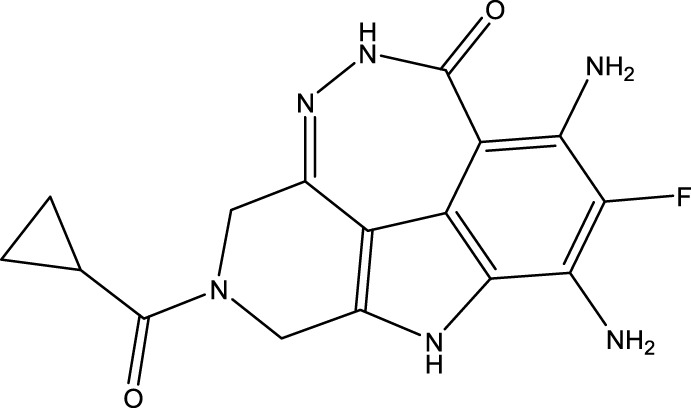	2.783
44b	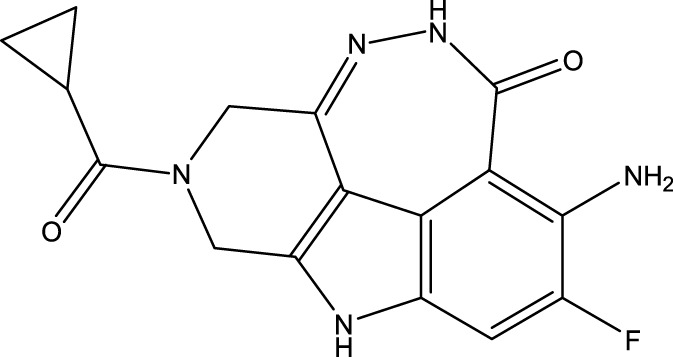	2.841
44c	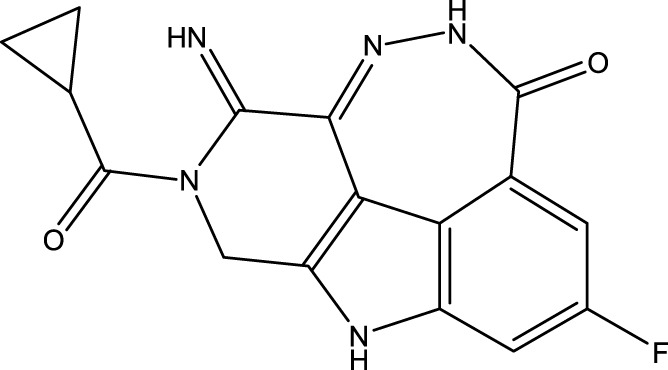	2.841
44d	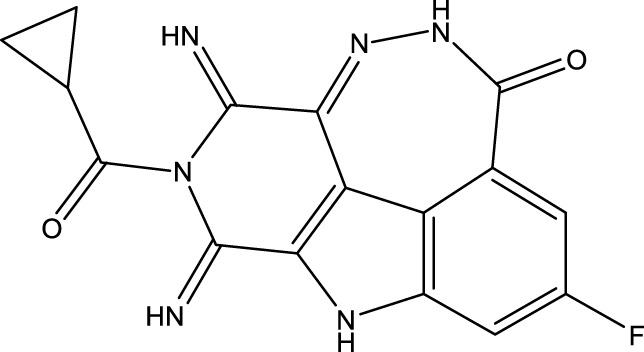	2.841
44e	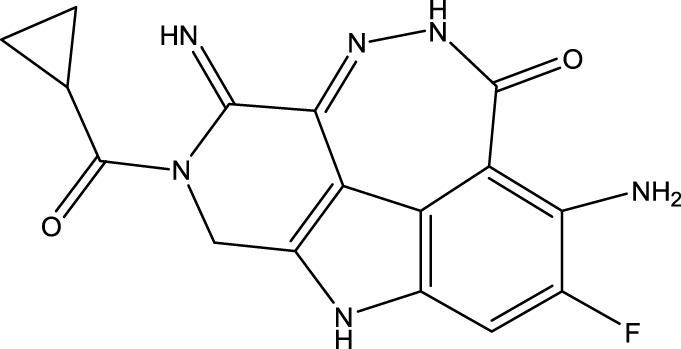	2.841
44f	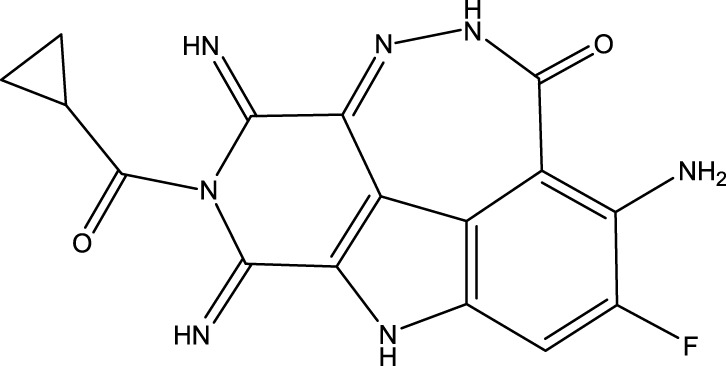	2.465

## 4 Comparison of different models

To verify the robustness of the models, k-fold cross-validation was used for model evaluation and the value of k was set to 5. All optimal predicted results of models based on HM, GEP, RF and SVR with single, double, and triple kernel function and their R^2^_cv_ are given in Table 10 respectively.

**TABLE 10:** Comparison of Statistical parameters of different methods.

Statistical parameters			HM	GEP	RF	SVR with single kernel function	SVR with double kernel function	SVR with triple kernel function
*R* ^2^	Training set		0.7550	0.7395	0.7503	0.9114	0.9259	0.9353
	Test set		0.9014	0.7818	0.8002	0.8599	0.9175	0.9348
RMSE	Training set		0.2327	0.2520	0.2378	0.0215	0.0180	0.0157
	Test set		0.2378	0.2768	0.212	0.0491	0.0289	0.0228
MAE	Training set		0.1991	0.2085	0.1518	0.1147	0.0973	0.0973
	Test set		0.1828	0.2039	0.0821	0.0928	0.0705	0.0745
R^2^_cv_	Training set		0.7773	0.7302	0.8701	0.8223	0.8989	0.9090
	Test set		0.6798	0.6998	0.8627	0.7815	0.8712	0.8971
Tetracyclic compounds	*R* ^2^		0.8417	0.8231	0.7202	0.9273	0.9493	0.9382
	RMSE		0.2342	0.2476	0.3078	0.1587	0.1325	0.1464
Pentacyclic compounds	*R* ^2^		0.9546	0.9291	0.9002	0.9702	0.9746	0.9766
	RMSE		0.2306	0.2884	0.342	0.1869	0.1727	0.1657
CCC	Training set		0.8833	0.8494	0.834	0.9259	0.9387	0.9336
	Test set		0.5017	0.42925	0.7839	0.757	0.8052	0.7992
Q2	Training set	Q^2^_F1_	0.9923	0.9919	0.9962	0.9976	0.998	0.9981
		Q^2^_F2_	0.829	0.7429	0.8849	0.9362	0.943	0.9494
	Test set	Q^2^_F1_	0.9934	0.9928	0.9991	0.9988	0.9991	0.9993
		Q^2^_F2_	0.2634	0.0259	0.8094	0.8697	0.8965	0.9178

It is obvious that the non-linear model established by SVR with triple kernel function shows the strongest prediction ability and model robustness than that of other models. Compared with the linear model built by HM, nonlinear models can better describe complicated problems. Compared with GEP which is easily trapped in the local optimal solution of the problem, SVR is a convex quadratic optimization method, which makes its local optimal solution the global optimal one. Compared to RF, the kernel function of SVR can be selected and adjusted according to the nature of the problem to adapt to different data distributions and patterns. Compared with SVR with single kernel function, the difference between *R* ^2^ of training set and test set in the model by SVR with double kernel function decrease from 0.0515 to 0.0084, which demonstrated the addition of polynomial kernel function did improve the generalization ability of SVR model. Compared with SVR with double kernel function, *R* ^2^ of training set and test set in the model by SVR with triple kernel function increased by 0.0094 and 0.0173, which demonstrated the addition of linear kernel function was helpful to improve the learning and generalization ability of SVR model.

Among the three models established by SVR with different numbers of kernel function, the model established by SVR with triple kernel function shows the best learning and generalization ability because kernel functions are complementary. In addition, the differences between the optimal *R* ^2^ and R^2^_cv_ of training set and test set in the model by SVR with triple kernel function are 0.0192 and 0.0309 which are much less than that of other models. It indicates that the model established by SVR with triple kernel function has strong robustness.

Furthermore, to verify the effect of compounds with different ring numbers on the predicted results, the original dataset was divided into tetracyclic compounds and pentacyclic compounds, and the *R* ^2^ and RMSE of six models related to these two compounds were calculated respectively. Table 10 shows that the prediction results by six models related to pentacyclic compounds better fit the measured values. *R* ^2^ of three SVR models related to tetracyclic compounds improve significantly. Furthermore, the improvement of SVR kernel function can better improve the performance of models related to tetracyclic compounds.

In addition, to demonstrate the external predictive ability of the model, Table 10 presents the calculation results of the three statistics Q^2^_F1_, Q^2^_F2_, and CCC under six different models.

## 5 Conclusion

In this study, 6 eight-parameter QSAR models were established by HM, GEP, RF and SVR with single, double, and triple kernel function to predict the biological activity of 57 FTPDDs as PARP1 inhibitors respectively. Compared with other models, the model established by SVR with triple kernel function shows the strongest prediction ability and robustness, which indicates that the method of SVR with triple kernel function has good potential for constructing models to predict the biological activity of compounds and guiding drug design. In addition, the PSO algorithm shows a strong parameter-optimized ability in the process of establishing SVR model due to its characteristic of high searching speed and fast convergence rate, which means PSO has good potential for optimizing parameters when building SVR model. Furthermore, the model established by SVR with triple kernel function shows 8 important factors that have a great influence on the biological activity of PARP1 inhibitors, which will guide new drug design and screening for breast cancer. Six FTPDDs were designed using these 8 important factors and molecular docking experiments were conducted on them. The properties of new derivatives were ultimately verified, and the effectiveness of the SVR model was also demonstrated.

## Data Availability

The original contributions presented in the study are included in the article/Supplementary Material, further inquiries can be directed to the corresponding author.
