# Supplementary material for: Study of PARP inhibitors for breast cancer based on enhanced multiple kernel function SVR with PSO
Source: Front Pharmacol. 2024 Feb 2;15:1257253. doi: 10.3389/fphar.2024.1257253 (PMC10869605; doi:10.3389/fphar.2024.1257253)
Supplement: Supplementary file 1 [file Table1.docx]

Supplementary Material

Table 1a. Measured and predicted lg (IC_50_) of FTPDDs 1- 16. *The compounds of the test set.

|  |  | | | | | | | | | | | |
| --- | --- | --- | --- | --- | --- | --- | --- | --- | --- | --- | --- | --- |
| Compound | R1 | R2 | R3 | A ring | Measured lg (IC_50_) |  | | Predicted lg (IC_50_) | | | | |
|  |  |  |  |  |  | HM | GEP | | RF | SVR with single kernel function | SVR with double kernel function | SVR with triple kernel function |
| 1* | H | H | H |  | 1.0086 | 1.0480 | 1.4606 | | 1.0899 | 1.2200 | 1.0728 | 1.2200 |
| 2 | H | F | H |  | 0.7782 | 1.0871 | 0.9972 | | 0.8096 | 0.9442 | 0.9517 | 0.9373 |
| 3 | H | H | F |  | 1.3802 | 1.2053 | 1.0629 | | 1.0685 | 1.3561 | 1.3675 | 1.3563 |
| 4 | H | F | H |  | 0.6902 | 0.7277 | 0.6955 | | 0.6945 | 0.6659 | 0.6774 | 0.6659 |
| 5* | H | H | H |  | 0.7993 | 0.7922 | 0.9886 | | 0.8630 | 0.8140 | 0.7881 | 0.8140 |
| 6 | H | F | H |  | 0.7160 | 0.9350 | 0.8954 | | 0.6831 | 0.8124 | 0.8201 | 0.7962 |
| 7 | F | H | H |  | 0.9590 | 1.0478 | 1.0962 | | 0.9185 | 0.9346 | 0.9459 | 0.9349 |
| 8 | H | F | H |  | 1.4594 | 1.1364 | 1.0550 | | 0.9721 | 1.0666 | 1.0749 | 1.0782 |
| 9* | H | F | H |  | 0.5185 | 0.7771 | 0.7436 | | 0.6377 | 0.6878 | 0.7119 | 0.6878 |
| 10 | H | F | H |  | 0.7634 | 0.6953 | 1.1288 | | 0.8384 | 0.7878 | 0.7760 | 0.7875 |
| 11 | H | F | H |  | 1.4082 | 1.1383 | 1.0468 | | 0.9584 | 1.1978 | 1.2552 | 1.2064 |
| 12* | H | H | H |  | 0.8261 | 0.8217 | 1.0543 | | 0.8586 | 0.9331 | 0.9033 | 0.9331 |
| 13* | H | F | H |  | 0.8129 | 0.6934 | 0.7544 | | 0.8265 | 0.6387 | 0.6627 | 0.6387 |
| 14 | H | F | H |  | 1.1173 | 1.0121 | 0.9203 | | 1.0431 | 1.1414 | 1.1301 | 1.0937 |
| 15 | H | F | H |  | 1.0569 | 1.1801 | 1.1249 | | 0.9004 | 1.0812 | 1.0693 | 1.0805 |
| 16 | H | F | H |  | 1.0253 | 0.7540 | 0.8502 | | 1.0659 | 1.0016 | 1.0128 | 1.0013 |

Table 1b. Measured and predicted lg (IC_50_) of FTPDDs 17- 34. *The compounds of the test set.

|  | |  | | | | | | | | | | |
| --- | --- | --- | --- | --- | --- | --- | --- | --- | --- | --- | --- | --- |
| Compound | R1 | | R2 | R3 | Measured lg (IC_50_) |  | | Predicted lg (IC_50_) | | | | |
|  |  |  |  |  |  | HM | GEP | | RF | SVR with single kernel function | SVR with double kernel function | SVR with triple kernel function |
| 17 | H | | H | CH_3_ | 1.1987 | 1.0851 | 0.9446 | | 1.2289 | 1.2230 | 1.2039 | 1.2223 |
| 18 | H | | CH3 | CH_3_ | 1.1818 | 0.9383 | 0.8455 | | 0.9208 | 1.1011 | 1.1694 | 1.1581 |
| 19 | H | | H | CH_2_CH_2_N(CH_3_)_2_ | 1.1761 | 1.1981 | 1.2457 | | 0.9703 | 1.2482 | 1.2250 | 1.2478 |
| 20 | H | | H | CH_2_CH_2_N(CH_2_CH_3_)_2_ | 1.1931 | 0.7624 | 0.7404 | | 1.0806 | 0.7965 | 0.8780 | 0.8462 |
| 21* | H | | H | CH_2_CH_2_NBn_2_ | 2.9031 | 2.7748 | 2.2265 | | 1.8054 | 2.3226 | 2.5306 | 2.3226 |
| 22 | H | | H |  | 1.0212 | 0.9255 | 0.7426 | | 0.9875 | 0.8878 | 1.0086 | 0.9971 |
| 23 | H | | H |  | 1.0128 | 1.0061 | 1.1865 | | 1.0120 | 0.9883 | 1.0005 | 0.9891 |
| 24 | H | | H |  | 2.5441 | 2.3479 | 2.4532 | | 2.4998 | 2.5196 | 2.3828 | 2.4649 |
| 25 | H | | CH3 | CH_2_CH_2_N(CH_3_)_2_ | 0.7404 | 0.8768 | 0.8938 | | 0.8139 | 0.8862 | 0.9145 | 0.8930 |
| 26 | F | | CH3 | CH_2_CH_3_N(CH_3_)_2_ | 0.8129 | 1.0090 | 0.7815 | | 0.7898 | 0.8372 | 0.8260 | 0.8372 |
| 27 | H | | CH3 | CH_2_CH_2_N(CH_2_CH_3_)_2_ | 0.5185 | 0.6547 | 0.8305 | | 0.8301 | 0.7285 | 0.6997 | 0.6425 |
| 28 | H | | CH3 | CH_2_CH_2_NBn_2_ | 2.7076 | 2.8163 | 2.6808 | | 2.3985 | 2.6835 | 2.6951 | 2.6842 |
| 29 | H | | CH3 |  | 0.8195 | 0.9021 | 0.8349 | | 0.8195 | 0.7954 | 0.8086 | 0.7961 |
| 30 | H | | CH_3_ |  | 0.4624 | 0.7450 | 0.8832 | | 0.7651 | 0.6989 | 0.7135 | 0.6598 |
| 31 | H | | CH_3_ |  | 2.2788 | 2.0614 | 2.1938 | | 2.2788 | 2.2725 | 2.2663 | 2.2547 |
| 32 | H | | CH_3_ |  | 1.3617 | 0.9713 | 1.0690 | | 1.2968 | 1.3373 | 1.3487 | 1.3377 |
| 33 | H | | CH_3_ |  | 1.4314 | 1.8920 | 1.0923 | | 1.4314 | 1.5439 | 1.4441 | 1.4552 |
| 34* | H | | CH_3_ | CH_2_CH_2_ NHCH_3_ | 0.8451 | 1.0300 | 0.9006 | | 0.8653 | 0.9870 | 1.0752 | 0.9870 |

Table 1c. Measured and predicted lg (IC_50_) of FTPDDs 35- 54. *The compounds of the test set.

|  | | |  | | | | | | | | |
| --- | --- | --- | --- | --- | --- | --- | --- | --- | --- | --- | --- |
| Compound | R1 | R2 | | Measured lg (IC_50_) |  | | Predicted lg (IC_50_) | | | | |
|  |  |  |  |  | HM | GEP | | RF | SVR with single kernel function | SVR with double kernel function | SVR with triple kernel function |
| 35 | H | H | | 1.1959 | 1.0914 | 1.1661 | | 1.0889 | 1.1718 | 1.1834 | 1.1725 |
| 36 | F | H | | 0.9294 | 0.7868 | 0.7288 | | 1.0242 | 0.8037 | 0.9158 | 0.9056 |
| 37 | F | CH_3_ | | 0.7559 | 1.0302 | 0.8291 | | 0.6576 | 0.7136 | 0.7432 | 0.7318 |
| 38 | F | CH_2_CH_3_ | | 0.6628 | 0.7414 | 0.7206 | | 0.6732 | 0.6867 | 0.7006 | 0.6943 |
| 39 | F | CH_2_CH_2_CH_3_ | | 0.6532 | 0.9517 | 0.8863 | | 0.6829 | 0.7932 | 0.7153 | 0.6835 |
| 40* | H | CH(CH_3_)_2_ | | 0.8513 | 0.9240 | 0.8963 | | 0.8273 | 0.7244 | 0.7829 | 0.7244 |
| 41 | F | CH(CH_3_)_2_ | | 0.7076 | 1.0675 | 0.8806 | | 0.7076 | 0.8558 | 0.8060 | 0.7999 |
| 42* | F | CH_2_ CH_2_CH_2_CH_3_ | | 0.5798 | 0.7356 | 0.8124 | | 0.6091 | 0.7469 | 0.7113 | 0.7469 |
| 43 | H |  | | 0.5185 | 0.7679 | 0.7753 | | 0.5479 | 0.5685 | 0.5860 | 0.5474 |
| 44 | F |  | | 0.3617 | 0.7052 | 0.6998 | | 0.3878 | 0.6200 | 0.5915 | 0.5470 |
| 45* | F |  | | 0.9912 | 0.8559 | 0.9505 | | 0.6812 | 0.9069 | 0.8375 | 0.9069 |
| 46 | F |  | | 1.4487 | 1.4786 | 1.5203 | | 1.3075 | 1.4730 | 1.4614 | 1.4723 |
| 47 | F |  | | 0.6721 | 1.1065 | 0.9807 | | 0.6588 | 0.6962 | 0.8079 | 0.6962 |
| 48 | F |  | | 1.2529 | 0.9978 | 1.0747 | | 1.0754 | 1.2285 | 1.2399 | 1.2286 |
| 49 | F |  | | 0.8633 | 0.6836 | 0.7265 | | 0.8156 | 0.8391 | 0.8508 | 0.8392 |
| 50 | F |  | | 0.9031 | 0.6838 | 0.7577 | | 0.7876 | 0.9277 | 0.9159 | 0.9267 |
| 51* | F |  | | 0.9956 | 0.5959 | 0.7650 | | 0.9328 | 0.9403 | 0.9290 | 0.9403 |
| 52 | H |  | | 0.8573 | 0.7521 | 0.7321 | | 0.8573 | 0.8336 | 0.8448 | 0.8334 |
| 53 | F |  | | 1.0792 | 0.7365 | 0.7139 | | 0.8423 | 1.0121 | 0.9618 | 0.9450 |
| 54* | F |  | | 0.8195 | 1.0923 | 0.8548 | | 0.5479 | 1.0716 | 1.0080 | 1.0716 |

Table 1d. Measured and predicted lg (IC_50_) of FTPDDs 55-57. *The compounds of the test set.

|  | |  | | | | | | | | | |
| --- | --- | --- | --- | --- | --- | --- | --- | --- | --- | --- | --- |
| Compound | R1 | | A ring | Measured lg (IC_50_) |  | | Predicted lg (IC_50_) | | | | |
|  |  |  |  |  | HM | GEP | | RF | SVR with single kernel function | SVR with double kernel function | SVR with triple kernel function |
| 55 | H | |  | 0.9138 | 0.9582 | 1.0044 | | 0.9069 | 0.8895 | 0.9262 | 0.9379 |
| 56 | F | |  | 0.7076 | 0.9856 | 0.8237 | | 0.7244 | 0.7324 | 0.7202 | 0.7316 |
| 57 | F | |  | 1.8633 | 1.3363 | 1.1642 | | 0.8030 | 1.3451 | 1.3750 | 1.4129 |

Table 2. The selected descriptors and their physical-chemical meanings and coefficient.

| Symbol | Physical-chemical Meaning | Coefficient |
| --- | --- | --- |
| MENANNB | Min e-n attraction for a N-N bond | 0.54386 |
| MEEROA | Min e-e repulsion for a O atom | -0.19081 |
| MRECCB | Max resonance energy for a C-C bond | 0.45861 |
| MBCM | Max bonding contribution of a MO | 7.2924 |
| ANRINA | Avg nucleoph. react. index for a N atom | -112.51 |
| KA(O3) | Kier shape index (order 3) | -0.58196 |
| HDH/T(QCP) | HA dependent HDCA-2/TMSA [Quantum-Chemical PC] | -192.22 |
| MRECHB | Max resonance energy for a C-H bond | -7.1692 |

Table 3. R^2^ matrix of the eight descriptors.

| Descriptor | MENANNB | MEEROA | MRECCB | MBCM | ANRINA | KA(O3) | HDH/T(QCP) | MRECHB |
| --- | --- | --- | --- | --- | --- | --- | --- | --- |
| MENANNB | 1.0000 |  |  |  |  |  |  |  |
| MEEROA | 0.0724 | 1.0000 |  |  |  |  |  |  |
| MRECCB | 0.0479 | -0.0651 | 1.0000 |  |  |  |  |  |
| MBCM | 0.5892 | 0.1741 | -0.0281 | 1.0000 |  |  |  |  |
| ANRINA | -0.1022 | -0.1146 | -0.3241 | 0.1031 | 1.0000 |  |  |  |
| KA(O3) | 0.5518 | -0.1184 | 0.2525 | 0.1826 | -0.5942 | 1.0000 |  |  |
| HDH/T(QCP) | -0.6837 | -0.0693 | 0.0308 | -0.5378 | -0.0850 | -0.5761 | 1.0000 |  |
| MRECHB | 0.3572 | 0.0891 | 0.1132 | 0.4526 | 0.0481 | -0.0158 | -0.1233 | 1.0000 |

Table 4. Predicted IC50 by HM and Docking total score of new FTPDDs

| No. | FTPDDs | Predicted IC_50_ | Total score |
| --- | --- | --- | --- |
| 44 |  | 0.7052 | 6.3154 |
| 44a |  | 0.5546 | 7.7065 |
| 44b |  | 0.5516 | 6.7748 |
| 44c |  | 0.5036 | 6.8673 |
| 44d |  | 0.4402 | 6.4417 |
| 44e |  | 0.3561 | 6.3002 |
| 44f |  | 0.3951 | 6.8984 |

Table 5. Predicted IC_50_ by HM and properties by PEA of newly designed compounds

| No. | Pre.IC_50_ | Toxicity | Logp | Solubility | Mol weight | TPSA | Drug likeness | Drug score |
| --- | --- | --- | --- | --- | --- | --- | --- | --- |
| 44 | 0.7052 | Medium | 1.75 | -4.13 | 326.0 | 77.56 | 6.72 | 0.63 |
| 44a | 0.5546 | Medium | -0.33 | -4.17 | 342.0 | 129.6 | 6.23 | 0.63 |
| 44b | 0.5516 | Medium | -0.35 | -4.09 | 327.0 | 103.5 | 6.32 | 0.64 |
| 44c | 0.5036 | Medium | -0.06 | -3.18 | 325.0 | 101.4 | 5.99 | 0.70 |
| 44d | 0.4402 | Medium | -0.17 | -2.49 | 338.0 | 126.2 | 5.05 | 0.72 |
| 44e | 0.3561 | Medium | -0.61 | -3.25 | 340.0 | 127.4 | 5.94 | 0.69 |
| 44f | 0.3951 | Medium | -0.84 | -2.56 | 353.0 | 151.2 | 4.99 | 0.71 |

Table 6. RMSD values between the selected docking pose of 7cmw and the experimental X-ray structure

| No. | FTPDDs | RMSD(A˚) |
| --- | --- | --- |
| 44 |  | 2.802 |
| 44a |  | 2.783 |
| 44b |  | 2.841 |
| 44c |  | 2.841 |
| 44d |  | 2.841 |
| 44e |  | 2.841 |
| 44f |  | 2.465 |

Table 7. Comparison of Statistical parameters of different methods

| Statistical parameters | | | HM | GEP | RF | SVR with single kernel function | SVR with double kernel function | **SVR with triple kernel function** |
| --- | --- | --- | --- | --- | --- | --- | --- | --- |
| R^2^ | Training set | | 0.7550 | 0.7395 | 0.7503 | 0.9114 | 0.9259 | 0.9353 |
|  | Test set | | 0.9014 | 0.7818 | 0.8002 | 0.8599 | 0.9175 | 0.9348 |
| RMSE | Training set | | 0.2327 | 0.2520 | 0.2378 | 0.0215 | 0.0180 | 0.0157 |
|  | Test set | | 0.2378 | 0.2768 | 0.212 | 0.0491 | 0.0289 | 0.0228 |
| MAE | Training set | | 0.1991 | 0.2085 | 0.1518 | 0.1147 | 0.0973 | 0.0973 |
|  | Test set | | 0.1828 | 0.2039 | 0.0821 | 0.0928 | 0.0705 | 0.0745 |
| R^2^_cv_ | Training set | | 0.7773 | 0.7302 | 0.8701 | 0.8223 | 0.8989 | 0.9090 |
|  | Test set | | 0.6798 | 0.6998 | 0.8627 | 0.7815 | 0.8712 | 0.8971 |
| Tetracyclic compounds | R^2^ | | 0.8417 | 0.8231 | 0.7202 | 0.9273 | 0.9493 | 0.9382 |
|  | RMSE | | 0.2342 | 0.2476 | 0.3078 | 0.1587 | 0.1325 | 0.1464 |
| Pentacyclic compounds | R^2^ | | 0.9546 | 0.9291 | 0.9002 | 0.9702 | 0.9746 | 0.9766 |
|  | RMSE | | 0.2306 | 0.2884 | 0.342 | 0.1869 | 0.1727 | 0.1657 |
| CCC | Training set | | 0.8833 | 0.8494 | 0.834 | 0.9259 | 0.9387 | 0.9336 |
|  | Test set | | 0.5017 | 0.42925 | 0.7839 | 0.757 | 0.8052 | 0.7992 |
| Q2 | Training set | Q^2^_F1_ | 0.9923 | 0.9919 | 0.9962 | 0.9976 | 0.998 | 0.9981 |
|  |  | Q^2^_F2_ | 0.829 | 0.7429 | 0.8849 | 0.9362 | 0.943 | 0.9494 |
|  | Test set | Q^2^_F1_ | 0.9934 | 0.9928 | 0.9991 | 0.9988 | 0.9991 | 0.9993 |
|  |  | Q^2^_F2_ | 0.2634 | 0.0259 | 0.8094 | 0.8697 | 0.8965 | 0.9178 |
