# Supplementary figures and images for: Study of PARP inhibitors for breast cancer based on enhanced multiple kernel function SVR with PSO
Source: Front Pharmacol. 2024 Feb 2;15:1257253. doi: 10.3389/fphar.2024.1257253 (PMC10869605; doi:10.3389/fphar.2024.1257253)

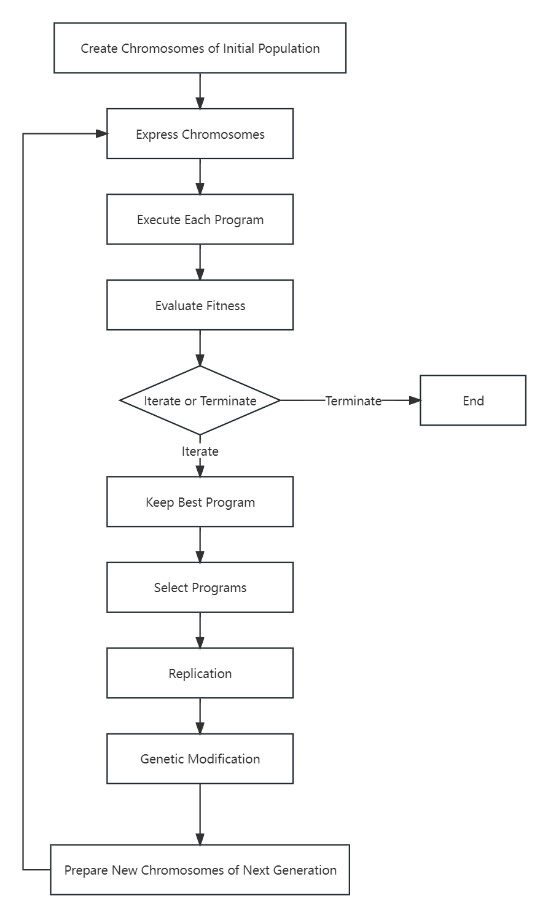

Supplement: Supplementary file 2 [file DataSheet1.ZIP › SupplementaryMaterial Presentation/Figure/Figure1.jpg]

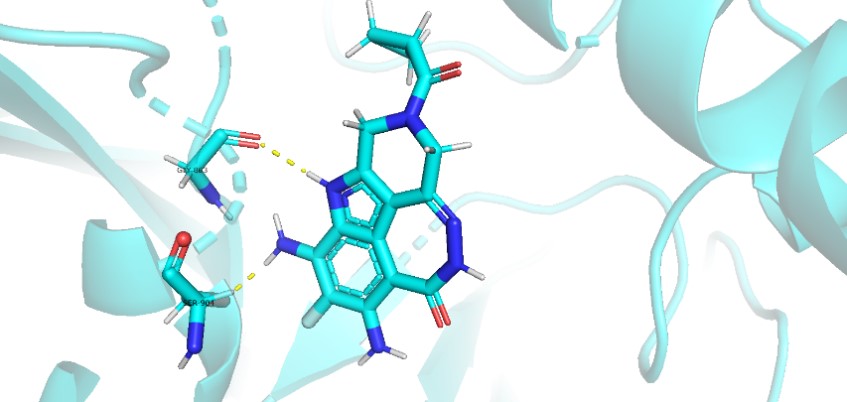

Supplement: Supplementary file 2 [file DataSheet1.ZIP › SupplementaryMaterial Presentation/Figure/Figure10.jpg]

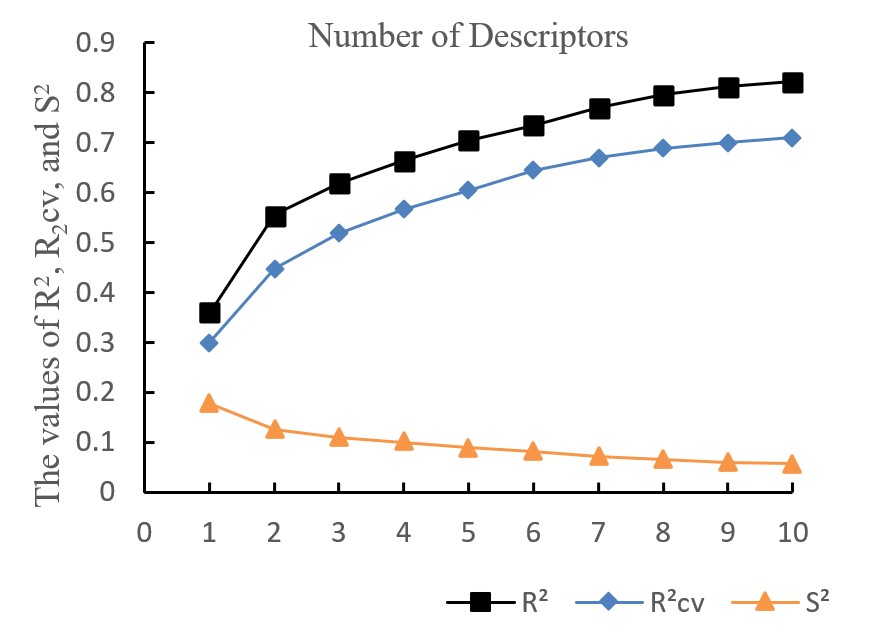

Supplement: Supplementary file 2 [file DataSheet1.ZIP › SupplementaryMaterial Presentation/Figure/Figure2.jpg]

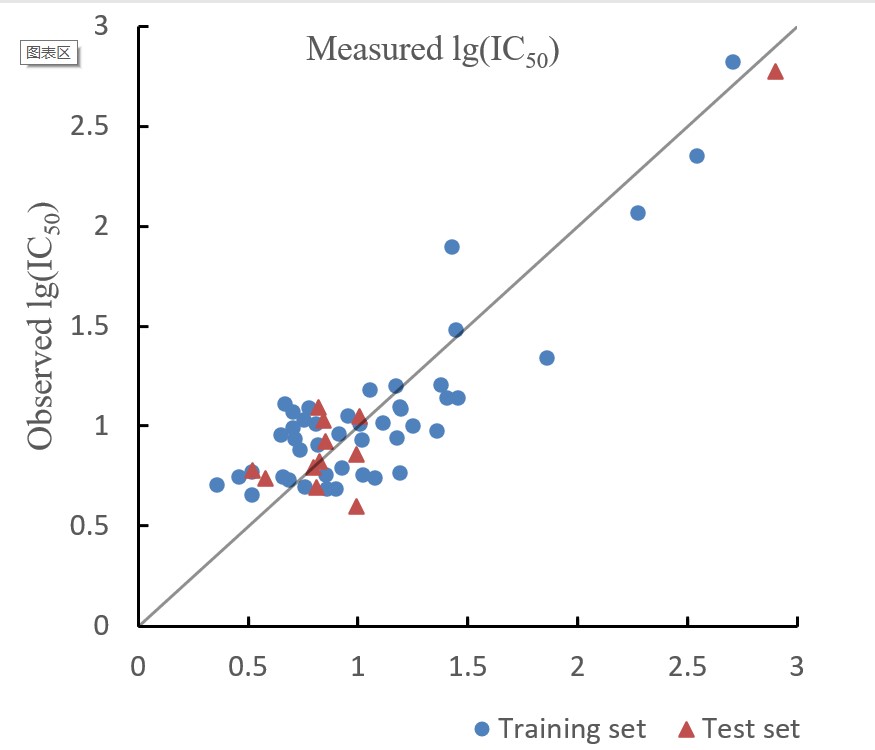

Supplement: Supplementary file 2 [file DataSheet1.ZIP › SupplementaryMaterial Presentation/Figure/Figure3.jpg]

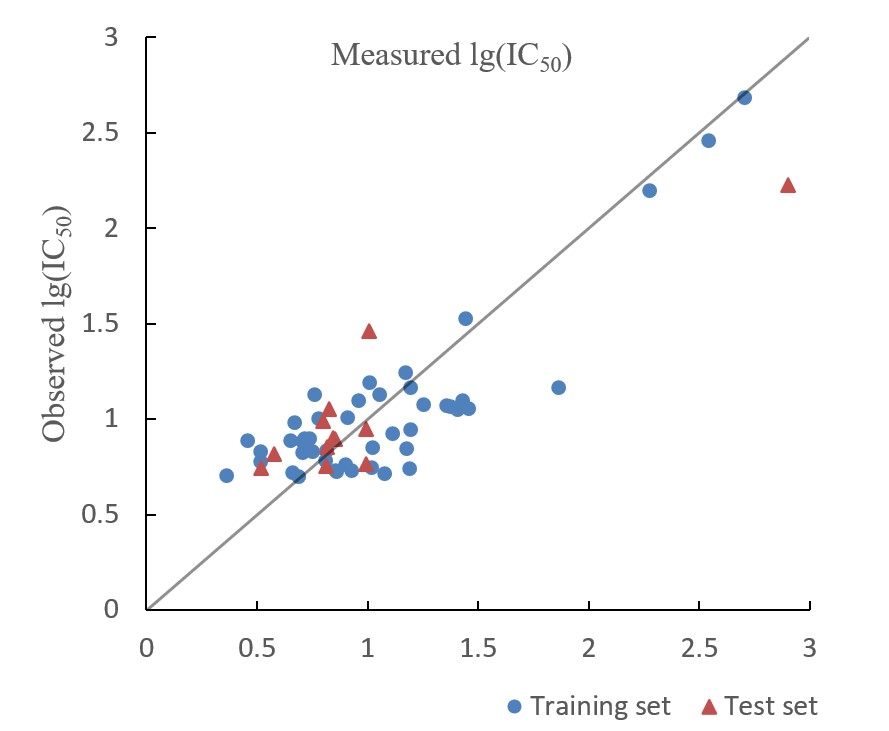

Supplement: Supplementary file 2 [file DataSheet1.ZIP › SupplementaryMaterial Presentation/Figure/Figure4.jpg]

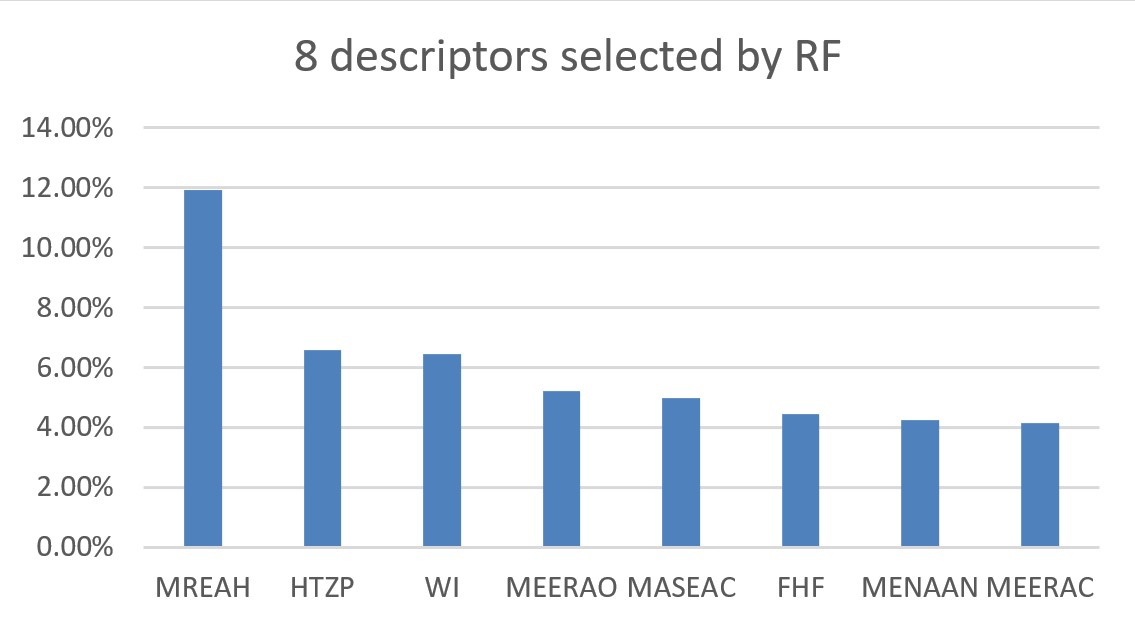

Supplement: Supplementary file 2 [file DataSheet1.ZIP › SupplementaryMaterial Presentation/Figure/Figure5.jpg]

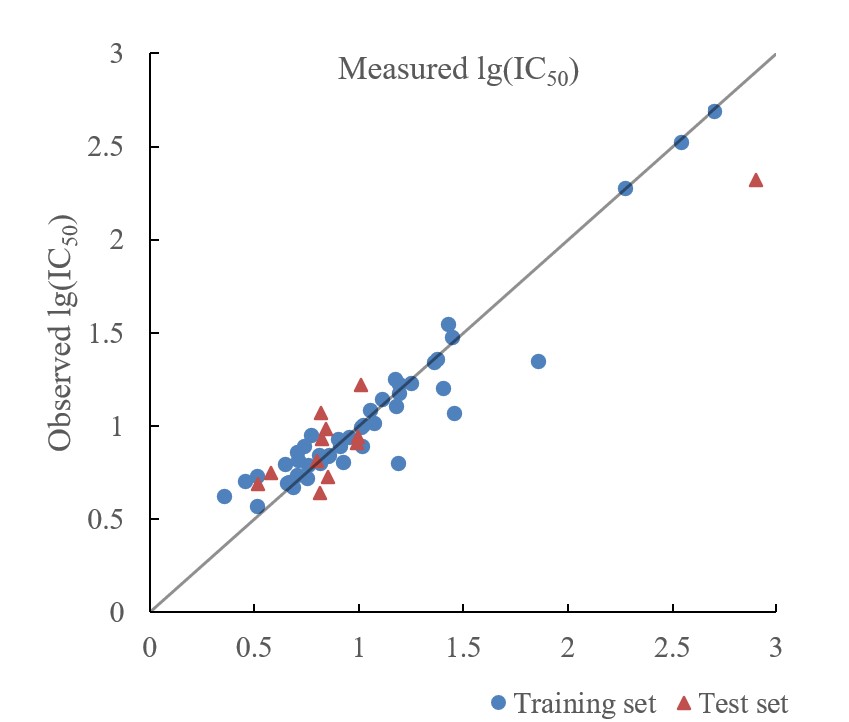

Supplement: Supplementary file 2 [file DataSheet1.ZIP › SupplementaryMaterial Presentation/Figure/Figure6.jpg]

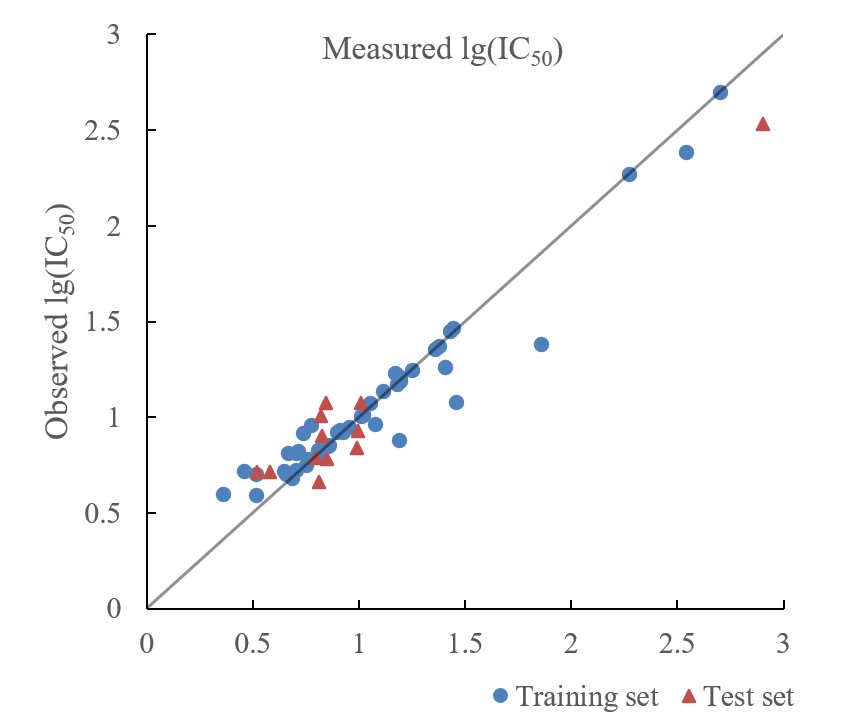

Supplement: Supplementary file 2 [file DataSheet1.ZIP › SupplementaryMaterial Presentation/Figure/Figure7.jpg]

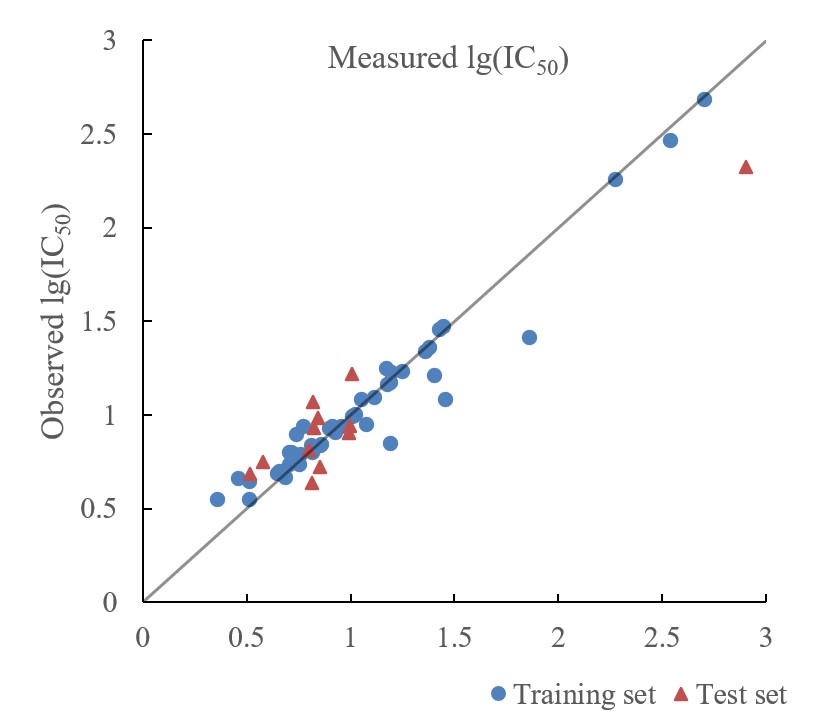

Supplement: Supplementary file 2 [file DataSheet1.ZIP › SupplementaryMaterial Presentation/Figure/Figure8.jpg]

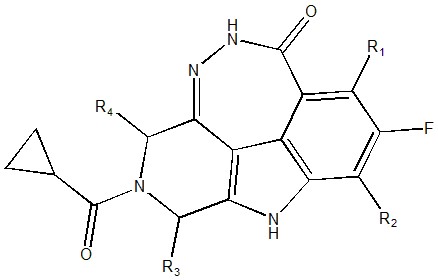

Supplement: Supplementary file 2 [file DataSheet1.ZIP › SupplementaryMaterial Presentation/Figure/Figure9.jpg]
